# The Relation Between eHealth Literacy and Online Health Information–Seeking Behavior: Systematic Review and Meta-Analysis

**DOI:** 10.2196/93578

**Published:** 2026-07-15

**Authors:** Xi Wang, Tian Shen, Xi Chen, Kejia He, Yuxiang Chris Zhao

**Affiliations:** 1 School of Information Management, Nanjing University, Nanjing, Jiangsu, China; 2 School of International Education, Nanjing University of Chinese Medicine, 138 Xianlin Ave, Nanjing, Jiangsu, 210023, China, 86 25 86562982; 3 School of Business, Nanjing University, Nanjing, Jiangsu, China

**Keywords:** digital health literacy, eHealth literacy, online health information seeking behavior, Generation Z, generational differences, meta-analysis

## Abstract

**Background:**

Online health information–seeking (OHIS) behavior shapes health self-management, and eHealth literacy—the ability to seek, appraise, and apply electronic health information—is regarded as its key driver. Previous reviews aggregated heterogeneous outcomes, focused on measurement properties, or examined single clinical populations, without isolating the eHealth literacy–OHIS link.

**Objective:**

This study quantified the strength and heterogeneity of the eHealth literacy–OHIS association and identified its boundary conditions across generation, morbidity status, and information source credibility.

**Methods:**

Following PRISMA (Preferred Reporting Items for Systematic Reviews and Meta-Analyses), we searched PubMed, Embase, Web of Science Core Collection, PsycINFO, Psychology and Behavioral Sciences Collection, and Library, Information Science, and Technology Abstracts (LISTA) up to March 15, 2026 (PROSPERO [International Prospective Register of Systematic Reviews] CRD420251088300). Eligible studies enrolled participants, measured eHealth literacy with validated instruments, and assessed OHIS. Risk of bias used the modified Newcastle-Ottawa Scale. Correlations were Fisher z–transformed and pooled under a random-effects model with the Hartung-Knapp-Sidik-Jonkman correction; subgroups were age cohort, morbidity status, and source type. Heterogeneity was quantified with *I*² and τ²; a univariate meta-regression examined temporal trends, and certainty of evidence was rated using GRADE (Grading of Recommendations, Assessment, Development, and Evaluation).

**Results:**

Of 9249 nonduplicate records, 32 studies entered the qualitative synthesis, and 19 (20 effect sizes) the meta-analysis. The grand mean correlation was 0.27 (95% CI 0.15-0.38; *P*<.001) but is of limited interpretive value given extreme heterogeneity (*I*²=99%; τ²=0.064; 95% prediction interval −0.26 to 0.67). Correlations were stronger in non–Gen Z (k=12; r=0.39; 95% CI 0.27-0.50; *P*<.001) than in Gen Z (k=8; r=0.07; 95% CI −0.06 to 0.20; *P*=.23), in patients (k=3; r=0.58; 95% CI 0.01-0.86; *P*=.049) than in nonpatients (k=17; r=0.22; 95% CI 0.11-0.32; *P*<.001), and in professional (k=5; r=0.41; 95% CI 0.11-0.64; *P*=.02) than in nonprofessional (k=14; r=0.21; 95% CI 0.06-0.35; *P*=.01) sources. Meta-regression on collection year showed no significant temporal change (b=−0.005 per year; *P*=.55), and neither the Egger test (*P*=.60) nor trim-and-fill indicated small-study effects.

**Conclusions:**

The eHealth literacy–OHIS association is best understood through its boundary conditions, not the overall estimate. The association was robust in non–Gen Z and professional-source contexts but near-null in Gen Z, showing that the eHealth literacy scale’s behavioral predictive validity is cohort- and platform-dependent. Interventions for Gen Z and nonpatient populations should pair literacy training with motivational cues and professionally curated information environments. GRADE certainty was very low, underscoring the need for longitudinal, performance-based research.

## Introduction

### Background

Digital media and communication technologies have markedly altered how people access health information, and many internet users have been reported to retrieve health-related information online [1]. Survey evidence has indicated widespread online health information seeking (OHIS) and self-diagnosis practices, and the internet has been shown to enable rapid retrieval of abundant and up-to-date material as well as bidirectional exchange between providers and consumers [2]. The affordances of social networking platforms, messaging services, video streaming, and intelligent conversational systems have further expanded opportunities for both one-to-many dissemination and many-to-many cocreation of health knowledge [3,4]. However, online health information originates from heterogeneous providers, and quality-control challenges persist, a situation that increases the risk of exposure to biased or inaccurate content [5,6].

OHIS behavior is a multifaceted construct encompassing dimensions such as thematic breadth, engagement depth, source selection, and search strategies [7]. Nevertheless, a significant portion of the literature has reduced this complexity to unidimensional metrics, such as search frequency or self-reported intensity [8], thereby oversimplifying the phenomenon and potentially masking the nuanced associations between eHealth literacy and search practices. Population-based surveys, for instance, often rely on frequency-based measures without distinguishing between preventive, diagnostic, or treatment-oriented inquiries [9]. Furthermore, while previous reviews have documented substantial heterogeneity in measurement frameworks, from self-efficacy-based instruments to performance-oriented assessments, there remains a critical gap in systematically linking these operational definitions to underlying cognitive mechanisms and observable behavioral outcomes. Such conceptual and methodological inconsistencies hinder a comprehensive understanding of how eHealth literacy correlates with distinct facets of OHIS, including information evaluation and application.

Prior research has identified significant generational variations in digital nativity and information-seeking preferences; whereas younger cohorts tend to exhibit more prevention-oriented behaviors, older adults often display lower objective literacy despite reporting higher perceived empowerment through digital engagement [9]. Generation Z (Gen Z) digital natives may possess strong technical skills, but their search behavior is often driven by intuition [10]. Furthermore, health status is closely associated with distinct information needs; patients and individuals with chronic conditions typically engage in more intensive searches across a diverse array of sources, yet they concurrently face an elevated risk of information overload [11]. Information source types also appear to interact with literacy levels, as individuals with higher eHealth literacy tend to prioritize professional portals and exercise more critical evaluation of social media content [12]. Meanwhile, the emergence of generative AI and chatbots has introduced new complexities regarding accessibility and information accuracy [13]. Despite the burgeoning literature, the theoretical pathways linking eHealth literacy to OHIS remain insufficiently delineated. While previous studies have established correlations between eHealth literacy and distal health outcomes such as medication adherence [14], the extent to which it relates to the frequency, scope, and selectivity of online searches remains unclear [7]. Specifically, the moderating roles of age, health status, and source characteristics have not been systematically quantified. Because a positive eHealth literacy–OHIS correlation is broadly expected, the key scientific value lies in identifying when and for whom this association weakens and what such attenuation implies for the validity of instruments such as the eHealth literacy scale (eHEALS) in contemporary digital environments.

Several systematic reviews have synthesized evidence on eHealth literacy, but their scope and analytical focus differ substantially from this study. One review [14] meta-analyzed the correlation between eHealth literacy and health-related behaviors (pooled *r*=0.31; n=14 studies), yet their outcome category subsumed health-promoting behaviors, medication adherence, and preventive actions as a whole, precluding any estimate specific to OHIS. Neter and Brainin [15] reviewed the literature on health literacy, eHealth literacy, and health outcomes among patients with long-term conditions, documenting associations with health service use and self-management, but did not pool effect sizes for information-seeking behavior or examine cross-population variability. Lee et al [7] systematically evaluated the psychometric properties of 7 eHealth literacy instruments using the Consensus-based Standards for the selection of health Measurement Instruments (COSMIN) methodology, confirming that eHEALS was the most widely validated scale while recommending content updates to reflect the evolving digital health landscape; their measurement-focused scope, however, did not extend to estimating how literacy levels relate to behavioral outcomes. Milanti et al [16] mapped the determinants and outcomes of eHealth literacy in healthy adults, identifying positive associations with general health-promotion behavior and health service use, yet treated information seeking only as an incidental component of broader outcome categories. Collectively, these reviews either aggregated heterogeneous behavioral outcomes without disaggregating information seeking, concentrated on measurement rather than behavioral correlates, or restricted their scope to specific clinical populations without cross-group comparisons. No prior synthesis has isolated the eHealth literacy–OHIS link as its primary focus, nor has any examined whether this association is moderated by generational cohort, morbidity status, or information source credibility. The present meta-analysis fills this gap.

### Objectives

This meta-analysis aimed to estimate the average association between eHealth literacy and OHIS and, more importantly, to quantify its heterogeneity across populations and information contexts. We specifically tested whether the association is attenuated in Gen Z and whether stronger health motivation and credible information cues amplify the translation of perceived literacy into active seeking.

## Methods

### Overview

**Figure 1.**
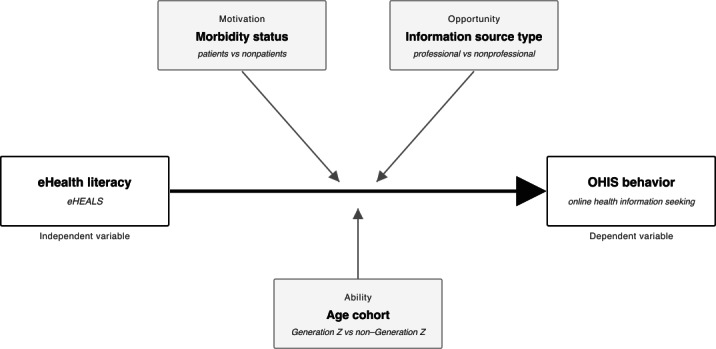
Motivation-Ability-Opportunity (MAO)–guided conceptual mapping of moderators in this meta-analysis. eHEALS: eHealth literacy scale; OHIS: online health information seeking.

Conceptually, the association between eHealth literacy and OHIS can be understood through the Motivation-Ability-Opportunity (MAO) framework (Figure 1), which also guided the selection of moderators in this meta-analysis. In this lens, eHealth literacy primarily reflects the perceived ability to locate and appraise online health information, whereas OHIS represents a coping-oriented behavior that is more likely to be enacted when health motivation is salient and when the information environment provides accessible and credible opportunities for seeking. Accordingly, a weak eHealth literacy–OHIS association may suggest that ability is no longer the primary constraint on behavior, or that commonly used self-report instruments capture general confidence rather than actionable competence in contemporary digital settings.

### Operational Definitions of Key Constructs

#### eHealth Literacy

eHealth literacy was originally conceptualized by Norman and Skinner [17] as the “Lily model,” describing an individual’s multifaceted ability to seek, comprehend, and critically appraise health information from electronic sources. The primary instrument used across the included studies was the eHEALS, which serves as a self-report measure of perceived competence in navigating and evaluating online health resources. Since its inception, eHEALS has demonstrated robust structural validity and internal consistency across diverse cultural and linguistic contexts [18]. In this meta-analysis, eHealth literacy was operationally defined as the aggregate score derived from validated measurement tools. To address potential measurement bias, we categorized these scores as indicators of perceived self-efficacy rather than objective technical skill, acknowledging the distinct psychological nature of self-reported literacy.

#### OHIS

Consistent with the conceptual framework proposed by Zimmerman and Shaw [19], OHIS is defined as the purposive ways in which individuals attempt to obtain information regarding health promotion, risk prevention, and disease management [20]. In the digital era, this theoretical construct manifests as an active acquisition process mediated by various platforms, including search engines, institutional portals, social media, and generative AI tools.

To resolve the inherent measurement heterogeneity across primary studies, we used a standardization protocol. All OHIS indicators were processed into continuous numerical values reflecting the magnitude of active search tendency. This approach ensures conceptual equivalence by focusing on the shared attribute of “active seeking,” regardless of whether the specific search objective was preventive, diagnostic, or treatment-oriented [21]. By standardizing these metrics, we enabled a robust comparison of effect sizes across heterogeneous research contexts and thematic scopes. Specifically, studies reporting OHIS on a continuous Likert scale or frequency scale were prioritized for direct use of their Pearson correlation coefficients with eHealth literacy. For studies reporting binary OHIS outcomes, the point-biserial correlation coefficient was extracted or computed from reported group statistics, which is mathematically equivalent to the Pearson *r* and can be directly included in the Fisher z meta-analysis [22]. Studies reporting only odds ratios or regression coefficients were converted to *r* using standard transformation formulas following the procedures described by Borenstein et al [23].

#### Literature Search

This systematic review and meta-analysis were conducted in strict adherence to the PRISMA-S (Preferred Reporting Items for Systematic Reviews and Meta-Analyses Literature Search Extension; Checklist 1) [24] and the PICO-SD (Population, Interest, Context, Outcome, and Study Design) framework. To ensure methodological transparency, the review protocol was prospectively registered in the PROSPERO (International Prospective Register of Systematic Reviews; registration ID: CRD420251088300). We searched PubMed (National Library of Medicine), Embase (Elsevier), Web of Science Core Collection (Clarivate), PsycINFO (EBSCOhost), Psychology and Behavioral Sciences Collection (EBSCOhost), and Library, Information Science, and Technology Abstracts (LISTA; EBSCOhost) to identify research papers published up to March 15, 2026. The search strategy used a Boolean logic framework structured around 4 primary concept blocks, including eHealth literacy, information-seeking behavior, online/digital contexts, and health-related terminology. To optimize sensitivity, we combined database-specific controlled vocabulary (eg, MeSH terms in PubMed and Emtree terms in Embase) with keywords and free-text terms using appropriate truncation and wildcards. The complete search syntax for each database, copied and pasted exactly as executed, is provided in Multimedia Appendix 1.

### Search Strategy

No date-of-publication limits were applied; each database was searched from inception. Searches were restricted to English-language publications because the validated eHealth literacy instruments central to this review (eg, eHEALS) have been studied predominantly in English-language literature, and the inclusion of non-English studies without access to validated translations could introduce measurement inconsistency. No published search filters (eg, study design filters) were applied.

To supplement the database searches, backward citation searching was performed by screening the reference lists of all included studies. We also searched Google Scholar; the first 200 results were screened by title. No study registries were searched, as this review focused on published observational studies rather than registered trials. No additional data were sought by contacting individual authors or experts.

The search strategies were developed de novo and peer reviewed by a second reviewer (TS) prior to execution, with reference to the Peer Review of Electronic Search Strategies (PRESS) guideline [25]. All retrieved records were imported into Zotero (Corporation for Digital Scholarship), and duplicates were removed using the software’s automated detection function followed by manual verification.

### Eligibility Criteria

For the systematic review, retrieved studies were selected according to the following inclusion criteria:

1. Population: individual-level participants, not health care professionals.
2. Exposure: eHealth literacy is measured using validated tools such as eHEALS, validated local versions, or clearly defined custom measures; it is measured as either continuous or categorical variables.
3. Comparator: no specific comparator.
4. Outcomes: active OHIS (binary, frequency, or Likert scale), related to health information only.
5. Study design: cross-sectional or longitudinal observational studies; mixed methods studies were included only when quantitative data were extractable.

Exclusion criteria were (1) studies that did not use validated or well-defined eHealth literacy measures; (2) qualitative research, review articles, or conference abstracts; (3) experimental studies where baseline observational data could not be isolated; (4) studies that did not report active OHIS; (5) non-English publications; and (6) studies where the full text was inaccessible.

### Selection Process

The literature search and selection process was performed independently by 2 reviewers (XW and Xiaohan Wang). Any discordance among the reviewers during the process of literature selection was resolved through mutual agreement or by involving a third researcher (XC) in a discussion. If 2 or more studies were performed on the same set of participants, the studies were considered duplicates, and only 1 comprehensive study was selected for further analysis.

### Data Collection Process

The following data were extracted from the selected literature using a standardized form by 2 reviewers (XW and Xiaohan Wang): the characteristics of the studies (first author, publication year, country or location, study design, participants, and sample size); types of eHealth literacy scales; mean eHealth literacy score; types of OHIS whose correlations with eHealth literacy were verified; methods of measuring OHIS; statistical analysis methods; types of outcome indicators; and values of outcome indicators. Any inconsistency or ambiguity was resolved by discussion with other reviewers (XC and TS).

### Study Risk of Bias Assessment

The risk of bias in the selected studies was assessed using the modified Newcastle-Ottawa Scale (NOS). While the NOS was developed for assessing the risk of bias in nonrandomized observational studies [26], our study used the modified NOS [27] that was developed for cross-sectional studies. The NOS uses a star system to assess the risk of bias in studies, whereby a lower score is associated with a higher risk of bias: high risk, 0‐3 stars; unclear risk, 4‐6 stars; and low risk, 7‐9 stars. Studies evaluated to have a high risk of bias were excluded from the meta-analysis.

### Qualitative and Quantitative Synthesis of the Results

#### Data Synthesis and Statistical Analysis

Given the substantial measurement heterogeneity identified across primary studies, diverse indicators of OHIS were conceptualized as a unified construct reflecting the intensity of active information acquisition. Rather than enforcing a rigid functional classification that might introduce conceptual ambiguity, the synthesis focused on the overall magnitude and direction of the association between eHealth literacy and the propensity to seek health information online.

For the quantitative synthesis, the pooled correlation coefficient was estimated via Fisher z transformation and subsequent inverse transformation to ensure the stability of the variance [23]. Correlation coefficients from individual studies were used as the primary unit of analysis, and multiple subgroup results from a single study were treated as independent observations to maximize data granularity. All included studies enrolled independent, nonoverlapping participant samples; no double-counting of participants occurred across effect sizes. The pooled estimates were reported with 95% CIs, and statistical significance was determined through rigorous hypothesis testing. The magnitude of the correlation coefficients was interpreted according to the benchmarks established in the existing psychological literature [28].

#### Heterogeneity and Bias Assessment

Cochran Q statistics and *I*² statistics were used to assess the degree of heterogeneity among the included studies. Given the anticipated diversity in study populations and measurement instruments, a random-effects model was prespecified to provide more conservative and generalizable estimates. This approach explicitly accounts for the variance both within and between studies [29]. The CIs for pooled estimates were derived using the Hartung-Knapp-Sidik-Jonkman correction, which produces more conservative interval estimates and is recommended for random-effects meta-analysis [30]. Potential small-study effects were evaluated using funnel plots and the Egger linear regression test, with the trim-and-fill method applied to estimate the impact of any missing studies [31].

#### Certainty of Evidence Assessment

The certainty of the evidence for each main outcome was evaluated using the GRADE (Grading of Recommendations, Assessment, Development, and Evaluation) framework [32]. For observational studies, the certainty rating starts at low and can be further downgraded based on 5 domains, including risk of bias, inconsistency, indirectness, imprecision, and publication bias. Upgrading was considered if a large effect size, a dose-response gradient, or plausible residual confounding was present. Two reviewers (XW and Xiaohan Wang) independently rated each domain, and disagreements were resolved through discussion with a third reviewer (XC). The results were summarized in a GRADE summary of findings table.

#### Subgroup and Sensitivity Analyses

To delineate the boundary conditions of the relationship between eHealth literacy and OHIS, 3 moderating factors were defined a priori based on the MAO framework. Age cohort was mapped to baseline digital opportunity and habitual access patterns; morbidity status reflected health motivation; and source type captured opportunity structures and credibility cues. The first distinction involved generational cohorts, distinguishing Gen Z from older cohorts to account for variations in digital fluency and information processing styles. Gen Z was operationally defined as individuals born between 1997 and 2012, consistent with widely cited demographic frameworks. Studies whose samples consisted exclusively or predominantly (≥80%) of participants within this birth cohort were coded as Gen Z; all others were coded as non–Gen Z. For studies reporting only mean age without a birth year range, samples with a mean age ≤26 years (ie, born ≥1997 at the time of data collection) were classified as Gen Z. Studies with mixed age distributions spanning both Gen Z and older cohorts, without stratified data, were assigned to non–Gen Z to avoid misclassification bias. The second distinction focused on health status, categorizing participants by morbidity status to reflect differences in health-related motivation and search urgency. Studies recruiting participants based on a confirmed clinical diagnosis or disease condition were classified as patients; studies drawing from community, student, or general population samples without a diagnostic inclusion criterion were classified as nonpatients. The third distinction concerned the type of information source, differentiating between professional portals and nonprofessional platforms to examine the impact of information credibility on user engagement. All statistical procedures were executed using R (version 4.5.1; R Foundation for Statistical Computing) with the meta and metafor packages.

## Results

### Study Selection

**Figure 2.**
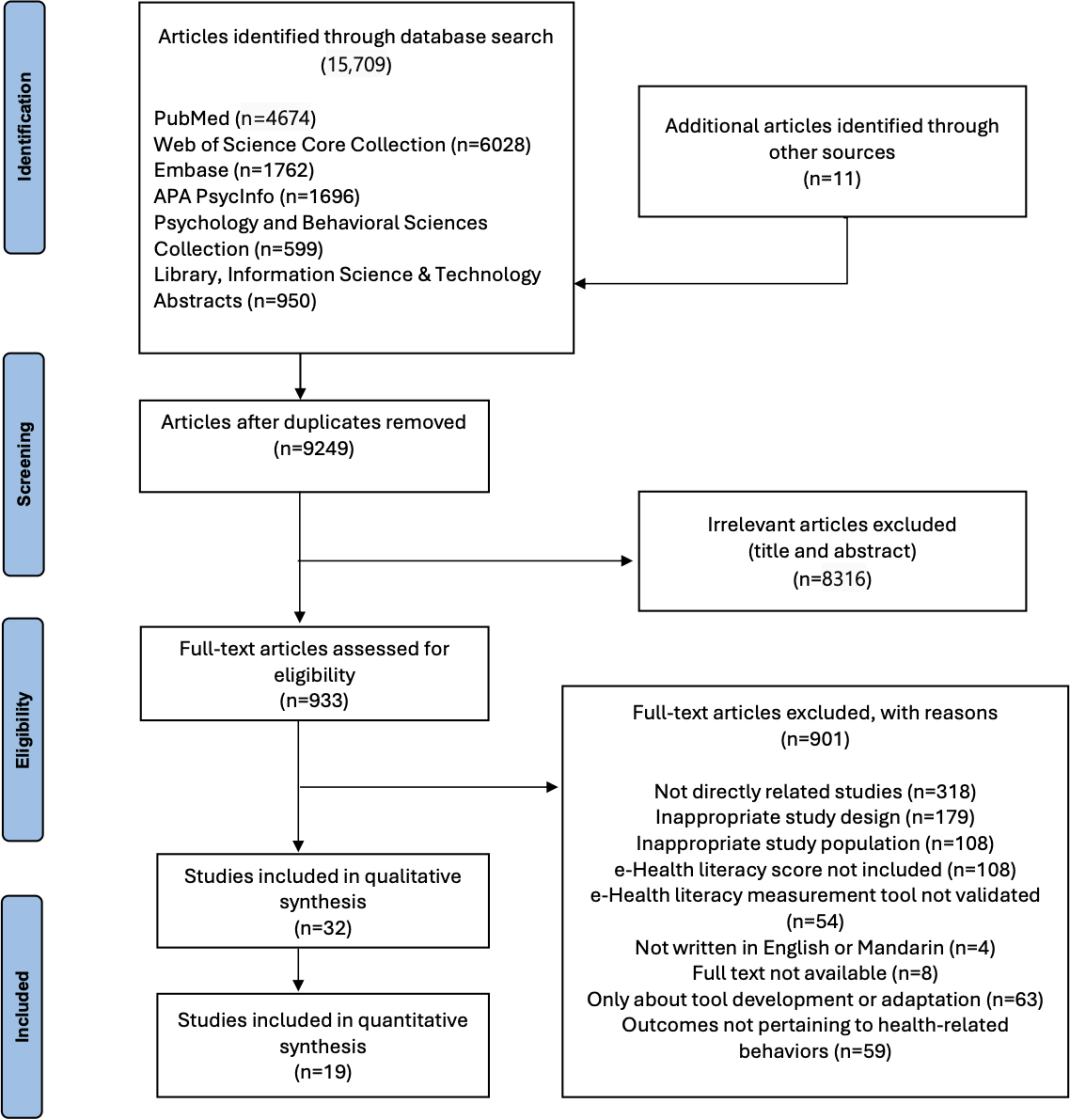
PRISMA (Preferred Reporting Items for Systematic Reviews and Meta-Analyses) 2020 flow diagram of study selection. Records were identified through database searches of PubMed, Embase, Web of Science Core Collection, PsycINFO, Psychology and Behavioral Sciences Collection, and Information Science and Technology Abstracts (LISTA; searched from inception to March 15, 2026).

Of 9249 identified nonduplicate studies, 8316 studies were excluded after a review of the studies’ titles and abstracts. The remaining 933 studies were assessed for eligibility through full-text review. Finally, 32 [33-64] studies, which presented the association between eHealth literacy and OHIS, were selected for qualitative analysis. Thirteen studies lacking sufficient data to compute correlations were excluded from the quantitative synthesis; among these, 2 studies also had a high risk of bias (NOS ≤3), resulting in 19 studies [33-51] included in the meta-analysis. A total of 19 eligible studies contributed 20 independent effect sizes, as one study reported separate subgroups analyzed independently. The detailed study selection process with the reasons for exclusion during screening steps is shown in Figure 2.

### Characteristics of the Included Studies

The overall characteristics of the included studies are summarized in Multimedia Appendix 2 [33-64]. Among the 32 studies, most were conducted in the United States [41,43,45,53,55,60,62] and China (n=7) [34,36,38,39,42,46,54], followed by Iran [56,61], Korea [47,58], and Serbia (n=2) [37,51]. The remaining studies were from Japan (n=1) [33], Austria (n=1) [52], Thailand (n=1) [57], Australia (n=1) [59], Bangladesh (n=1) [40], the United Kingdom (n=1) [44], Indonesia (n=1) [63], Norway (n=1) [64], Poland (n=1) [48], Denmark (n=1) [49], and Brazil (n=1) [50]. Additionally, one [35] study was an international collaboration involving multiple countries. Most studies were conducted in Asia. The majority of studies (n=28) were cross-sectional investigations using questionnaires, while 2 studies implemented mixed methods frameworks [33,52]. The age groups of the study participants varied: adolescents [37,46,50-52], college or university students [36,38,40,44,56,63], and older adults [41,47,62]. Nine [39,42,45,49,55,59-61,64] studies focused on patients or clinical groups, including those with chronic obstructive pulmonary disease [60], stroke [39], various chronic conditions [59], physical disabilities [55], type 2 diabetes [61], and patients in primary care [42], otolaryngology clinics [45], or after coronary interventions [49,64].

Among the 32 included studies, 31 used the original eHEALS or a country-specific language or culturally adapted version. Versions of the eHEALS were adapted into Japanese, Korean, Chinese, Serbian, Indonesian, Persian, and Brazilian Portuguese. One study used the Polish version of the Transactional e-Health Literacy Instrument [48]. Characteristics of the OHIS observed across the included studies are summarized in Multimedia Appendix 2. Preventive information seeking was most often conducted through high-reach and low-threshold sources such as search engines, public or government health portals, news aggregators, and widely used social or video platforms, indicating that content related to lifestyle guidance, nutrition, physical activity, and vaccination is typically consumed within mainstream and multimedia environments. Diagnosis- and treatment-related information seeking was concentrated in more authoritative sources, including hospital and professional society websites, national health portals, encyclopedic health resources, and online medical service platforms, reflecting a stronger demand for credibility and clinical specificity at the point of illness or decision-making. Self-management information seeking gravitated toward interactive and longitudinal platforms such as online support groups, patient forums, disease-specific apps, subscription or call-center services, and—emerging in the most recent studies—conversational agents and large language model–based chatbots, underscoring a shift from one-time retrieval to ongoing, peer-supported engagement in chronic care. Across all 3 dimensions, marked heterogeneity in source preference was evident by age group, health status, and digital experience. Adolescent and student samples concentrated their preventive and diagnostic searches within short video, microblogging, and mobile-first environments, whereas older adults and patient cohorts emphasized official portals, structured apps, and desktop-based access for self-management and treatment information. This generational layering of digital health practices, visible across multiple countries, suggests that the adoption of social and AI-enabled platforms does not occur uniformly but reflects differing needs and affordances.

### Risk of Bias Assessment

The risk of bias assessment revealed that 11 studies [37,39,41,42,45-48,51,60,61] had a low risk of bias, and 19 studies [33-36,38,40,43,44,49,50,52-57,59,62,64] had an unclear risk of bias. Two studies [56,58] were found to have a high risk of bias with a NOS score of 3, exhibiting significant selection biases in population representativeness and sample size calculation, alongside failure to control confounding variables and use validated tools for measuring OHIS (Multimedia Appendix 3).

### Qualitative Analysis of eHealth Literacy and OHIS Behavior

Among the 32 included studies, 24 [33-43,45-47,50,52,54-56,58,61-64] reported significant positive associations between eHealth literacy and OHIS, 4 [44,49,53,57] reported no significant associations, 2 [48,60] reported mixed or dimension-specific findings, and 2 [51,59] reported inverse associations.

The null results tended to arise in studies with narrow outcomes or small samples and in analyses treating eHealth literacy as a mediator or moderator rather than a direct predictor [44,49,53,57]. Mixed and dimension-specific patterns highlighted substantial heterogeneity. For example, one study using a multidimensional instrument demonstrated that functional, communicative, and critical eHealth literacy could be associated with different health services in opposite directions [48]. Another study documented that eHealth literacy was significantly and positively correlated with most types of OHIS except for nutrition information seeking and online health product purchasing [60]. The 2 inverse associations were observed in a chronic-condition sample, suggesting possible reverse causation or compensatory information-seeking behavior [59], and in an adolescent sample, where higher eHealth literacy was associated with lower likelihood of seeking mental health information online [51].

Across 24 studies [33-43,45-47,50,52,54-56,58,61-64] reporting positive associations, higher eHealth literacy was most consistently linked to greater search frequency, use of a wider range of sources, more active cross-checking, increased adoption of health portals and mobile apps, participation in online support communities, and targeted searches related to prevention, diagnosis, and self-management. Objective measurement studies using screen recordings or browser logs corroborated these self-reported patterns and documented richer and more diverse search behaviors among participants with higher literacy levels [44,58]. One study explicitly examined large language model–based chatbots and conversational assistants as information sources and found that higher eHealth literacy predicted both greater use of these AI-mediated tools and more frequent follow-up cross-checking after chatbot interactions [35]. However, studies explicitly involving AI-mediated information sources remained limited, and generalization of these findings should be cautious. The relationship between eHealth literacy and OHIS in the included studies is summarized in Multimedia Appendix 4.

### Quantitative Analysis of eHealth Literacy and OHIS Behavior

#### Correlation Between eHealth Literacy and OHIS by Population Characteristics and Sources

The correlation coefficients between eHealth literacy and OHIS in the studies [33-51] included in the meta-analysis ranged from −0.33 to 0.72. Among these studies, the correlation between eHealth literacy and OHIS was synthesized by age, morbidity status, and sources of OHIS. From the age subgroups, the pooled estimates of correlation coefficients were 0.07 (95% CI −0.06 to 0.20) for Gen Z participants and 0.39 (95% CI 0.27‐0.50) for non–Gen Z participants. For morbidity status, the pooled correlation coefficient of the patient group was 0.58 (95% CI 0.01‐0.86), whereas that of the nonpatient group was 0.22 (95% CI 0.11‐0.32). Regarding the sources of OHIS, professional sources yielded a higher pooled correlation coefficient (0.41, 95% CI 0.11‐0.64) compared with nonprofessional sources (0.21, 95% CI 0.06‐0.35; Table 1).

Sensitivity analysis revealed that the correlation in the patient subgroup remained stable even after excluding any single study, confirming the preliminary robustness of this finding despite the small number of studies [39,45,49].

**Table 1.** Pooled correlation coefficients (*r*) and 95% CIs between eHealth literacy and online health information seeking (OHIS) behavior by population subgroup, estimated using random-effects meta-analysis applied to cross-sectional studies conducted in 12 countries.

Characteristic	Studies, n (%)	Pooled correlation coefficient^a^ (95% CI)
Age		
Gen Z^b^	8 (40)	0.07 (–0.06 to 0.20)
non–Gen Z	12 (60)	0.39 (0.27 to 0.50)
Morbidity status		
Patients	3 (15)	0.58 (0.01 to 0.86)
Nonpatients	17 (85)	0.22 (0.11 to 0.32)
Sources		
Professional	5 (26)	0.41 (0.11 to 0.64)
Nonprofessional	14 (74)	0.21 (0.06 to 0.35)

a Correlation coefficients were transformed using Fisher z and pooled with a random-effects model.

b Gen Z: generation Z.

#### Overall Estimate of the Correlation Between eHealth Literacy and OHIS Behavior

**Figure 3.**
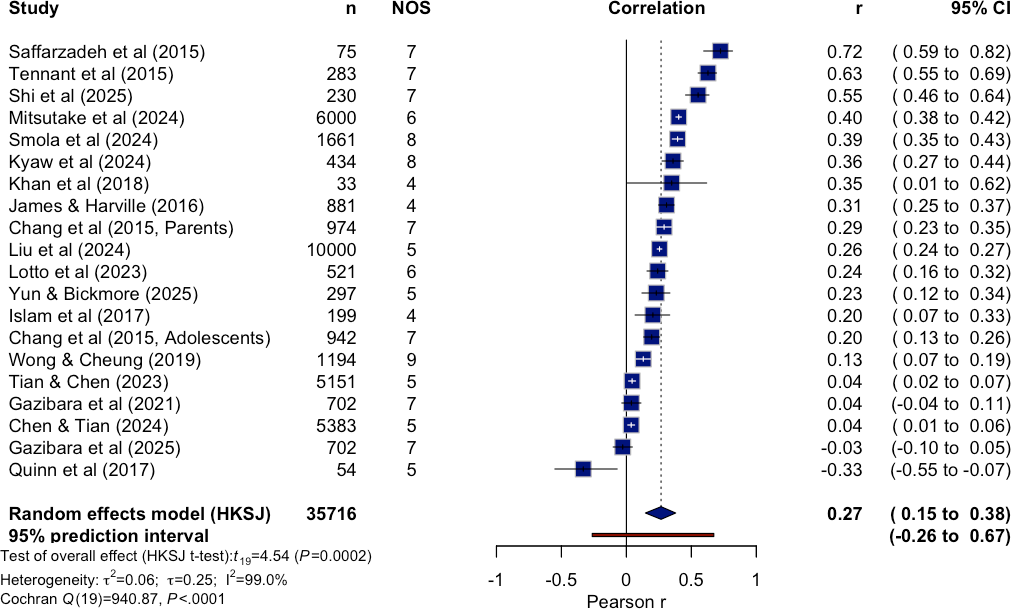
Forest plot of Pearson correlation coefficients (*r*) between eHealth literacy and online health information seeking (OHIS) behavior across 19 [33-51] cross-sectional observational studies. HKSJ: Hartung-Knapp-Sidik-Jonkman; NOS: Newcastle-Ottawa Scale.

**Figure 4.**
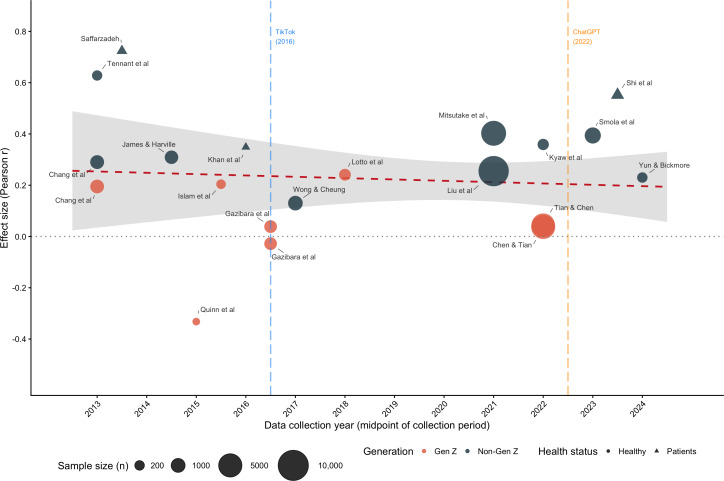
Effect size (Pearson r) plotted against data collection year (midpoint) for 20 effect sizes from 19 [33-51] studies.

The overall estimate of the correlation between eHealth literacy and OHIS was conducted for 19 studies [33-51] contributing 20 effect sizes available for quantitative analysis. The pooled correlation coefficient was 0.27 (95% CI 0.15‐0.38; *P*<.001), with high heterogeneity (Cochran Q_19_=940.87; *P*<.001; *I*²=99%), which indicated a small-to-moderate correlation [28] between eHealth literacy and OHIS (Figure 3). However, the effect was highly heterogeneous and nearly absent in Gen Z (*r*=0.07) while substantially stronger among patients (*r*=0.58). Given the substantial heterogeneity observed, we additionally conducted sensitivity analyses and subgroup examinations to explore potential sources of variation. Heterogeneity likely reflects differences in population age, measurement tools, and operational definitions of OHIS across studies. Given the *I*² value of 99.0%, the assumption of a single underlying effect size is untenable; instead, the pooled *r*=0.27 should be read as the grand mean of a distribution of context-specific effects rather than a point estimate applicable to any individual setting. The wide 95% prediction interval (−0.26 to 0.67) reinforces this interpretation: in some contexts the association is negligible or even negative, whereas in others it is moderate to large. We therefore foreground the boundary conditions revealed by subgroup and temporal analyses as the primary narrative of this meta-analysis, rather than the pooled average itself. The 95% prediction interval for the overall association ranged from −0.26 to 0.67, spanning negligible to large positive effects and thus confirming that the eHealth literacy–OHIS relationship is substantially context-dependent. Prediction intervals for the subgroups are reported in Table 1. Notably, the prediction interval for the patient subgroup (−0.45 to 0.95) was the widest, reflecting the heterogeneous nature of “patient” populations across included studies, whereas the Gen Z prediction interval (−0.25 to 0.38) was narrower and centered near zero, consistent with the attenuated average effect in this cohort. To explore whether the eHealth literacy–OHIS association has weakened over time as digital environments become more algorithmically driven, we conducted a univariate meta-regression with data collection year (midpoint) as a moderator, displayed as a bubble plot in which each bubble is sized by study weight (Figure 4). The meta-regression yielded a slope of b=−0.005 per year (95% CI −0.022 to 0.012; *P*=.55), indicating a nonsignificant negative temporal trend across the full sample. However, the nonsignificance may reflect competing dynamics: among Gen Z studies, effect sizes clustered near zero regardless of collection year, whereas among non–Gen Z studies, effect sizes remained moderate to large across the observation window (2013‐2024). Thus, the temporal attenuation appears largely attributable to the growing share of Gen Z–focused studies in more recent years rather than a uniform secular decline.

**Figure 5.**
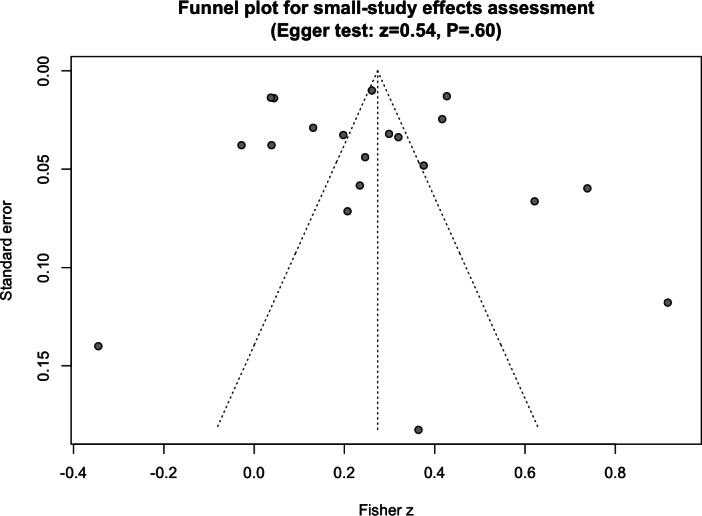
Funnel plot of standard error against Fisher z-transformed correlation coefficient (*r*) for 20 effect sizes from 19 studies examining the association between eHealth literacy and online health information seeking (OHIS) behavior.

Visual inspection of the funnel plot (Figure 5) revealed minor asymmetry; however, the Egger regression test did not yield statistically significant results (z=0.54; *P*=.60), indicating no evidence of small-study effects. Subsequent analysis using the trim-and-fill method identified no missing studies requiring imputation to restore symmetry, and the pooled effect size remained unchanged following adjustment. These findings suggest that the meta-analytic results are robust and unlikely to be substantially influenced by small-study effects, including potential publication bias.

To evaluate the robustness of the findings, influence diagnostics and leave-one-out analyses were performed. The results showed no single study had an unduly large impact on the overall pooled correlation coefficient. The influence diagnostics plot is provided in Multimedia Appendix 5.

Two additional sensitivity analyses were conducted to address potential sources of bias. First, to evaluate the impact of study quality on the pooled estimate, we conducted sensitivity analyses at progressively higher NOS thresholds. All 20 effect sizes in the quantitative synthesis came from studies scoring NOS≥4. When restricted to studies scoring NOS≥6 (k=12), the pooled correlation was *r*=0.34 (95% CI 0.18‐0.49); when further restricted to NOS≥7 (k=10), the estimate was *r*=0.35 (95% CI 0.14‐0.52). These results suggest that the overall finding was not driven by lower-quality studies; rather, higher-quality studies tended to yield slightly larger effect sizes. Two additional studies in the qualitative synthesis received NOS scores of 3 [56,58]; however, neither reported a quantifiable effect size suitable for meta-analytic pooling, so their absence from the quantitative synthesis reflects insufficient statistical data rather than quality-based exclusion.

Second, to address the concern that treating multiple effect sizes from the same study as independent observations may inflate precision, we reran the overall analysis using only one effect size per study (selecting the effect size with the largest sample or, when tied, the first reported). This one-per-study analysis (k=19) yielded a pooled *r*=0.28 (95% CI 0.15‐0.39), consistent with the primary result, suggesting that the inclusion of 2 within-study estimates from Chang et al [46] did not substantially alter the conclusions.

The GRADE assessment of certainty of evidence is provided in Multimedia Appendix 6. The certainty was rated very low for all outcomes (overall and every subgroup), reflecting 2 levels of downgrading from the low starting level (cross-sectional studies): one level for serious inconsistency (*I*²≥78.5% in all analyses) and one level for imprecision (all 95% prediction intervals crossed zero and spanned from negligible to moderate or large effect sizes). No downgrading was applied for risk of bias, indirectness, or publication bias.

## Discussion

### Prior Work and Principal Findings

OHIS is a central component of contemporary health self-management [11,12], and eHealth literacy is often considered a key determinant of how individuals engage with online health resources [16,50]. However, whether eHEALS meaningfully predicts OHIS across contemporary cohorts and platforms remains unclear. Previous studies have shown that higher eHealth literacy tends to correlate with more frequent and effective health information–seeking behavior, particularly in health promotion and disease prevention [14,15]. However, the relationship between eHealth literacy and health behavior is not always straightforward [41]. Studies suggest that individuals with higher eHealth literacy are generally more engaged in OHIS, such as physical activity and disease prevention, but these behaviors may vary based on factors like age and health status [51].

Moreover, while frequent engagement with health information online is common, it is not always linked to positive health outcomes [11]. For example, despite a higher frequency of online health information searching, individuals with lower eHealth literacy may struggle to critically assess the quality of the information they access, which can result in misinformed decisions or health behavior reluctance [52,53]. Older adults and those with chronic conditions may face additional barriers when navigating online health resources, even if they engage with health information more often [14]. Our subgroup results indicate that age and morbidity status are not merely covariates but boundary conditions that reshape the literacy-behavior link.

Our analysis showed an overall positive relationship between eHealth literacy and various types of OHIS. Across most of the included studies, higher eHEALS scores were significantly associated with more frequent or more diverse OHIS, including preventive information, diagnosis- and treatment-related topics, and self-management behavior. This positive pattern was consistent across different cultural and linguistic adaptations of the eHEALS [18]. However, a few studies reported mixed or even negative findings. For example, Lee et al [59] reported nonsignificant or weak associations between eHealth literacy and the perceived ease of searching, suggesting contextual variations, and Quinn et al [44] reported that eHEALS was weakly negatively correlated with the search difficulty score. Therefore, the effect of eHealth literacy on OHIS should be carefully interpreted, and the possibility of other factors mediating the relationship should be considered. As eHealth literacy alone cannot explain the correlation between online health information acquisition and OHIS, additional research is needed to identify other factors [14,54]. The GRADE assessment rated the certainty of this evidence as very low, primarily because all included studies were cross-sectional in design and heterogeneity remained extreme across all analyses. This rating is typical of correlational meta-analyses in the eHealth literacy field, where observational designs predominate, and it highlights the importance of interpreting the pooled estimates as indicative trends rather than precise quantifications [14,16]. Accordingly, the subgroup patterns identified in this review should be regarded as hypothesis-generating findings that require confirmation through longitudinal and performance-based research.

### Generational Variations in the Association Between eHealth Literacy and OHIS Behavior

Our analysis showed that the association between eHealth literacy and OHIS was notably stronger among non–Gen Z participants than among Gen Z. This finding is consistent with prior research indicating that, although younger cohorts often report higher self-rated digital health literacy and use online resources more frequently, their predictive value for actual health-related behaviors is weaker [9,10]. Jiao et al [54] found that while Chinese Gen Z respondents were active in seeking online health information, digital health literacy explained less variance in their OHIS compared with older groups. Similarly, Papp-Zipernovszky et al [10] reported that in Hungary, despite older adults scoring lower on the eHEALS, eHealth literacy was a stronger predictor of health empowerment and behavior. Together with these studies, our results suggest that in non–Gen Z populations, eHealth literacy is more effectively translated into health-related actions, whereas in Gen Z, high digital familiarity may dilute the extent to which eHealth literacy influences actual health behaviors.

A particularly notable finding is the near-null association in Gen Z (*r*=0.07). From the perspective of the MAO framework, this attenuation may indicate that perceived ability is no longer the primary constraint for digital natives; instead, motivation and opportunity structures may play a dominant role in determining whether and how they seek health information [65]. Moreover, eHEALS primarily captures confidence in locating and evaluating information within a Web 1.0 and Web 2.0 paradigm, such as searching and judging websites [17], while Gen Z increasingly acquires health information through algorithmically curated feeds, short-video platforms, and AI-mediated interfaces [13,35]. Under these conditions, eHEALS may serve less as a behavioral predictor and more as a general self-efficacy indicator, raising concerns about its construct validity in Web 3.0 environments [7,8]. This cognitive distinction also suggests that next-generation eHealth literacy frameworks should incorporate algorithmic literacy and heuristic resistance, particularly as search behaviors shift toward AI-mediated and conversational systems [35].

### Health Status and Motivational Activation of eHealth Literacy

The stronger correlation between eHealth literacy and OHIS in patients likely results from the combination of higher health information needs and regular health care interactions, which make their eHealth literacy more directly applicable to seeking relevant information [11,15]. Health-related motivations, such as disease symptoms or treatment concerns, activate patients’ eHealth literacy, enabling them to effectively use online resources [11]. Health care providers also play a significant role by guiding patients to trusted online resources, enhancing the application of eHealth literacy in information seeking related to self-management [21]. In contrast, nonpatients—while still seeking preventive information or lifestyle advice—often lack the immediate health concerns that would activate their eHealth literacy in a similar way [16]. Their skills remain underused without the context of health-related urgency or professional prompts, limiting their engagement in OHIS [15]. This suggests that interventions for nonpatients should not only focus on building eHealth literacy but also incorporate cues that activate information-seeking behaviors. It should be noted, however, that the patient subgroup was itself heterogeneous with respect to disease type and severity. The included patient-population studies encompassed distinct clinical contexts: stroke inpatients in a rehabilitation setting (*r*=0.55, n=230) [39], otorhinolaryngology outpatients with mixed chronic and acute conditions (*r*=0.72, n=75) [45], and patients who underwent cardiac surgery during early recovery (*r*=0.35, n=33) [49]. Although all 3 yielded above-average correlations, the conditions ranged from chronic neurological disease to acute postoperative recovery, and sample sizes were small (total n=338). With only 3 studies and no within-subgroup variance in disease severity coding, formal meta-regression by disease chronicity or severity was not feasible. We note descriptively that the 2 studies involving patients with chronic or chronic-plus-acute conditions produced larger effects than the purely acute postoperative sample, consistent with the MAO framework prediction that sustained health motivation strengthens the literacy–behavior link. Future research should examine whether disease chronicity, illness severity, or treatment stage systematically moderates the eHealth literacy–OHIS association within patient populations.

### Role of Information Source Credibility in Shaping OHIS Behavior

Professional sources carry institutional credibility and structured cues that lower the effort of evaluating and verifying information, so eHealth literacy translates more directly into purposeful OHIS [6,59]. Literate users can follow clinician-recommended portals or authoritative institutional pages and reach high-quality answers quickly, which strengthens the observed association [12,59]. These sources also embed prompts and pathways that orient searches toward actionable information and increase perceived usefulness [66]. In contrast, nonprofessional venues emphasize peer narratives, algorithmic feeds, and social endorsement; even highly literate users often enter them for social or experiential reasons rather than to obtain verified solutions, which weakens the direct literacy-seeking link for authoritative content [4,54]. Interventions should therefore combine eHealth literacy training with an information environment that offers clear provenance, clinician referrals, and low-friction access to vetted portals to source skills into searches with stronger health impact [66].

### Strengths and Limitations

This study systematically reviewed and synthesized research on the relationship between eHealth literacy and OHIS. By quantifying the pooled effect size across different populations, illness statuses, and sources, it offers a comprehensive evidence base for understanding how individual eHealth literacy shapes both the choice of information sources and the quality of retrieval [14,15]. Subgroup analyses further revealed that correlations were stronger when information was obtained from professional sources than from nonprofessional ones, highlighting the role of literacy in navigating credible online resources. These findings provide a data-driven foundation for designing targeted digital health interventions and tailoring eHealth services to different user groups. More importantly, the pronounced attenuation in Gen Z raises the hypothesis that the predictive validity of eHEALS may be cohort- and platform-dependent, although this finding should be interpreted with caution given the cross-sectional, self-report nature of the underlying data.

However, several limitations should be considered when interpreting these findings, which can be categorized across 3 distinct levels. At the measurement level, virtually all included studies relied on eHEALS as a perceived self-efficacy instrument, meaning the construct captured is “perceived ability” rather than objective or performance-based eHealth literacy; this distinction is important because the 2 may relate differently to actual information-seeking behavior [7,58]. OHIS was similarly operationalized through self-report, and the lack of behavioral or log-based measures precludes verification of actual search activity [44]. At the study design level, the predominance of cross-sectional designs prevents any assessment of temporal dynamics, directionality, or change in the eHealth literacy–OHIS relationship over time [23]; longitudinal and experimental designs are needed to establish causal ordering. At the broader conceptual level, the boundary between eHealth literacy as a predictor versus a moderator of OHIS remains undertheorized, and the role of contextual factors such as device access, internet speed, and platform affordances was rarely controlled for in primary studies, potentially confounding the observed associations [4,16]. Additionally, the GRADE certainty of evidence was very low for all outcomes, indicating that future research with stronger designs is very likely to alter the estimated associations. This underscores the need for prospective cohort studies to produce more definitive evidence. Although eHEALS was developed in the early Web era [17], the observed generational attenuation may itself be interpreted as evidence that the instrument’s behavioral predictive validity is context-dependent rather than universally stable. Future eHealth literacy measurement frameworks should consider incorporating next-generation competencies, such as algorithm awareness (the capacity to recognize and critically evaluate algorithmic content curation) and heuristic resistance (the ability to identify and counteract cognitive shortcuts exploited by platform design), alongside traditional self-efficacy constructs, to better capture health information navigation behaviors in AI-mediated and algorithmically curated digital environments [35].

### Conclusion

The eHealth literacy–OHIS association is best understood through its boundary conditions rather than a single pooled estimate. The link was robust in non–Gen Z and professional-source contexts but near-null in Gen Z, suggesting that eHEALS, originally developed for Web 1.0/2.0 search settings, captures behaviorally relevant competence unevenly across cohorts and platforms [9,10,17]. In contrast to prior reviews that aggregated heterogeneous outcomes, focused on measurement properties, or examined single clinical populations, this synthesis isolates the eHealth literacy–OHIS link and quantifies its boundary conditions across generation, morbidity, and source credibility within one framework. For policy and practice, interventions targeting Gen Z and nonpatient groups should pair literacy training with motivational cues and access to credible, professionally curated portals, rather than relying on literacy training alone [21,66]. Given very low GRADE certainty and the cross-sectional, self-report nature of available evidence, longitudinal and performance-based studies—together with algorithm-aware extensions of eHEALS for AI-mediated and conversational platforms—are needed to clarify causal direction in the Web 3.0 era [35].

## Supplementary material

10.2196/93578 Multimedia Appendix 1 Search strategy.

10.2196/93578 Multimedia Appendix 2 Characteristics of the included studies.

10.2196/93578 Multimedia Appendix 3 Risk of bias assessment for the included studies using the modified Newcastle-Ottawa Scale.

10.2196/93578 Multimedia Appendix 4 The relationship between eHealth literacy and online health information seeking behaviors in the included studies.

10.2196/93578 Multimedia Appendix 5 The influence diagnostics plot.

10.2196/93578 Multimedia Appendix 6 GRADE summary of findings for the association between eHealth literacy and online health information seeking behavior.

10.2196/93578 Multimedia Appendix 7 Open dataset underlying the meta-analysis.

10.2196/93578 Checklist 1 PRISMA 2020 expanded checklist.

## Abbreviations

COSMIN: Consensus-based Standards for the selection of health Measurement Instruments
eHEALS: electronic health literacy scale
Gen Z: generation Z
GRADE: Grading of Recommendations, Assessment, Development, and Evaluation
LISTA: Library, Information Science, and Technology Abstracts
MAO: Motivation-Ability-Opportunity
NOS: Newcastle-Ottawa Scale
OHIS: online health information seeking
PICO-SD: Population, Interest, Context, Outcome, and Study Design
PRESS: Peer Review of Electronic Search Strategies
PRISMA-S: Preferred Reporting Items for Systematic Reviews and Meta-Analyses Literature Search Extension
PROSPERO: International Prospective Register of Systematic Reviews
