# Supplementary material for: The Relation Between eHealth Literacy and Online Health Information–Seeking Behavior: Systematic Review and Meta-Analysis
Source: J Med Internet Res. 2026 Jul 15;28:e93578. doi: 10.2196/93578 (PMC13372218; doi:10.2196/93578)
Supplement: Multimedia Appendix 1 [file jmir-v28-e93578-s001.docx]

**Multimedia Appendix 1. Search strategy.**

| **Database** | **Search Fields / Syntax** | **Search String** |
| --- | --- | --- |
| **PubMed** | MeSH Terms + Title/Abstract | (("Health Literacy"[Mesh] OR "eHealth literacy"[tiab] OR "electronic health literacy"[tiab] OR "digital health literacy"[tiab] OR "internet health literacy"[tiab] OR "online health literacy"[tiab] OR "web-based health literacy"[tiab] OR "digital literacy"[tiab] OR "health literacy"[tiab])AND("Information Seeking Behavior"[Mesh] OR "information seeking"[tiab] OR "information behavior"[tiab] OR "information use"[tiab] OR "information access"[tiab] OR "information retrieval"[tiab] OR seek*[tiab] OR search*[tiab] OR find*[tiab] OR retriev*[tiab] OR access*[tiab])  AND(internet[tiab] OR online[tiab] OR web[tiab] OR "social media"[tiab] OR "digital platform"[tiab] OR "digital environment"[tiab])AND(health[tiab] OR medical[tiab] OR medicine[tiab] OR wellness[tiab] OR illness[tiab] OR healthcare[tiab] OR patient[tiab])) |
| **Web of Science Core Collection** | Topic (TS) | TS = ( ("eHealth literacy" OR "electronic health literacy" OR "digital health literacy" OR "internet health literacy" OR "online health literacy" OR "web-based health literacy" OR "health literacy" OR "digital literacy") AND ("information seeking" OR "information behavior" OR "information use" OR "information access" OR "information retrieval" OR seek* OR search* OR find* OR retriev* OR access*) AND (internet OR online OR web OR "social media" OR "digital platform" OR "digital environment") AND (health OR medical OR medicine OR wellness OR illness OR healthcare OR patient) ) |
| **Embase** | Emtree Terms + Free Text | ('ehealth literacy'/exp OR 'ehealth literacy' OR 'electronic health literacy' OR 'digital health literacy' OR 'internet health literacy' OR 'online health literacy'/exp OR 'online health literacy' OR 'web based health literacy' OR 'digital literacy'/exp OR 'digital literacy' OR 'health literacy'/exp OR 'health literacy') AND ('information seeking behavior'/exp OR 'information seeking behavior' OR 'information seeking'/exp OR 'information seeking' OR 'health information seeking' OR 'online health information seeking' OR 'internet health information' OR 'web based health information seeking' OR 'health information search' OR 'health information behavior' OR 'information use' OR 'information access'/exp OR 'information access' OR 'internet use'/exp OR 'internet use' OR 'web use'/exp OR 'web use') AND ('internet'/exp OR internet OR 'online'/exp OR online OR 'web'/exp OR web OR 'social media'/exp OR 'social media' OR 'digital platform'/exp OR 'digital platform' OR 'digital environment') AND ('health'/exp OR health OR medical OR 'medicine'/exp OR medicine OR 'wellness'/exp OR wellness OR 'illness'/exp OR illness OR 'healthcare'/exp OR healthcare OR 'patient'/exp OR patient) |
| **APA PsycInfo**  **Psychology and Behavioral Sciences Collection**  **Library, Information Science & Technology Abstracts** | Abstract | ("eHealth literacy" OR "electronic health literacy" OR "digital health literacy" OR "internet health literacy" OR "online health literacy" OR "web-based health literacy" OR "health literacy" OR "digital literacy")  AND("information seeking" OR "information behavior" OR "information use" OR "information access" OR "information retrieval" OR seek* OR search* OR find* OR retriev* OR access*)AND(internet OR online OR web OR "social media" OR "digital platform" OR "digital environment")AND(health OR medical OR medicine OR wellness OR illness OR healthcare OR patient) |
