# Supplementary material for: The Relation Between eHealth Literacy and Online Health Information–Seeking Behavior: Systematic Review and Meta-Analysis
Source: J Med Internet Res. 2026 Jul 15;28:e93578. doi: 10.2196/93578 (PMC13372218; doi:10.2196/93578)
Supplement: Multimedia Appendix 2 [file jmir-v28-e93578-s002.docx]

**Multimedia Appendix 2.** Characteristics of the included studies.

| Author (year) | Country | Population (sample n) | eHealth literacy measurement scale | OHIS behaviors measurement tool | Types of   OHIS sources | Risk of bias |
| --- | --- | --- | --- | --- | --- | --- |
| Mitsutake et al (2024) [33] | Japan | Adults internet users 20 to 79 years (n=6000) | Japanese version of eHEALS | 110-item list of web sources+ 10-topic search checklist | Search engines, news portals, public sites, Social media sites, YouTube, blogs, Health-related Q&A sites, physician sites | 6 |
| Maitz et al (2020) [52] | Austria | Students aged 12 to 14 years (n = 14) | eHEALS | Analyze search query frequency and webpage access metrics | classification system of Pérez to classify search engines. | 4 |
| Pho et al (2022) [53] | United States | TGD and cisgender sexual minority adults (n=3258) | eHEALS | 23 items from the adapted HINTS | Social networking sites,  e-mail or Internet to communicate with doctor, | 5 |
| Liu et al (2024) [34] | China | Chinese internet users aged ≥18 (n=10,000) | Adapted eHEALS | Frequency of OHIS | mainstream media, professional health media, aggregator news platforms, web portals, open forums, online support forums, search engines, and individual social media account | 5 |
| Yun and Bickmore (2025) [35] | International (US, UK, Canada, Australia, South Africa, Poland, etc.) | Online crowd workers(n=297) | eHEALS | Web-based survey questionnaire developed based on literature review | Search engines, health-related websites, social media, health community forums, LLM-based chatbots,health apps, conversational assistants | 5 |
| Jiao et al (2023) [54] | China | Chinese respondents aged 18+(n=1,862) | eHEALS | Adapted HINTS | search engines, social media | 5 |
| Robinson-Whelen et al (2023) [55] | United States | Women with physical disabilities who used the Internet (n=508) | eHEALS | HINTS | social media sites,health-related video | 4 |
| Khademian et al [56] | Iran | College students from Shiraz University of Medical Sciences (n=386) | Adapted eHEALS | frequency of internet use for specific health items | Search engines, mobile apps , online social network, blogs, official health organization websites, internet magazines | 3 |
| Tian and Chen (2023) [36] | China | Chinese university students aged 18 to 22 years (n=5151) | Chinese version of eHEALS | Self-developed OHIS behaviors scale | social media | 5 |
| Htet et al  (2023) [57] | Thailand | Myanmar migrants in Hat Yai & Pattani (n=1050) | eHEALS | A question with “Yes” or “No” answer | websites, Facebook, YouTube, Viber/Line, SMS subscription, call center, mobile health apps | 5 |
| Kim et al (2020) [58] | Korea | Adults who searched for information ≥5 times/week  (n=31) | Korean version of eHEALS | Real-time screen recording | Naver, Daum, Google, blogs, café websites, news portals, encyclopedias, Korean National Cancer Information Center | 3 |
| Gazibara et al (2021) [37] | Serbia | High school students in central Belgrade (n=702) | Serbian version of eHEALS | Frequency scale (never, rarely, sometimes, often) via single-item question | YouTube, social networks, health forums, health blogs, websites run by doctors, websites run by health institutions | 7 |
| Chen and Tian (2024) [38] | China | university students (n = 5,383) | Chinese version of eHEALS | Online questionnaires of OHIS behaviors | Websites, social media, health-specific sites | 5 |
| Shi et al  (2025) [39] | China | Young and middle-aged stroke patients (n=230) | eHEALS | scale developed by Yoon and Kim | Search engines, Professional medical websites, Official health websites, Social media, Online medical service platforms | 7 |
| Lee et al  (2015) [59] | Australia | Adults with chronic health conditions (n=400) | eHEALS | Study-specific questionnaire | Search engines, Public sites (e.g., government/professional), Social media sites | 6 |
| Islam et al  (2017) [40] | Bangladesh | University students aged 17-35 years (n=199) | eHEALS | Survey on Web 2.0 use | Popular social media, Web-based support groups, Blogs | 4 |
| Tennant et al (2015) [41] | United States | Baby boomers and older adults using the Internet (n = 283) | eHEALS | HINTS items on electronic device use and Web 2.0 for health information | Facebook, Twitter, LinkedIn | 7 |
| Stellefson et al. (2018) [60] | United States | COPD patients from COPD Foundation’s National Research Registry (n = 176) | eHEALS | HINTS items on electronic device use and social media use for COPD-related health information | social networking sites, discussion boards, blogs | 7 |
| Wong and Cheung (2019) [42] | China (Hong Kong) | Primary-care clinic patients (n = 1194) | eHEALS | 25-item self-administered questionnaire covering frequency, contents, sources, reasons, and patterns of online health information seeking | Wikipedia, health portals, medical encyclopedias, Q&A sites, hospital or clinic websites | 9 |
| James and Harville (2016) [43] | United States (Florida) | African American adults (n = 881) | eHEALS | Self-administered questionnaire covering sources of health information and topics searched online in the past 12 months | Internet, smartphone apps,television | 4 |
| Peimani et al (2024) [61] | Iran | Individuals with type 2 diabetes mellitus (n=1143) | Persian version of eHEALS | 3-item measure | Online support groups,  Internet | 8 |
| Xie B (2011) [62] | United States | Older adults aged 52 to 91 (n=172) | eHEALS | Modified items from Kaiser Family Foundation and PEW Internet | NIHSeniorHealth.gov, MedlinePlus.gov | 6 |
| Quinn et al  (2017) [44] | United Kingdom(Northern Ireland) | University students and staff (n=54) | eHEALS | Browser-based tracking software | Search engine; Government websites; Academic websites; Commercial websites; | 5 |
| Puspita et al (2024) [63] | Indonesia | Pharmacy students at two pharmacy diploma schools (n=314) | Indonesian version of eHEALS | Semi-structured questionnaire | Instagram, TikTok, YouTube, WhatsApp, Twitter | 5 |
| Saffarzadeh (2015) [45] | United States | Adult outpatients visiting a tertiary care otolaryngology clinic (n=75) | eHEALS | Adapted Pew Health Online Survey questions | Dedicated health sites, University/Hospital sites, Professional society sites, Search engines, Social media, YouTube, Wikipedia | 7 |
| Chang et al (2015) [46] | China | Junior high school students and their parents in Taiwan (Students n=1,869; Parents n=1,365) | eHEALS | 5-point scale | Government websites, Healthcare websites, Nonprofit organization websites, Commercial websites | 7 |
| Kyaw et al. (2024) [47] | South Korea | Community-dwelling older adults aged 65+ years (n=434) | eHEALS | 5-point Likert scale for frequency of internet | Social media platforms prevalent in South Korea | 8 |
| Ramstad et al. (2023) [64] | Norway | Adults after percutaneous coronary intervention (n=1970) | eHEALS | Study-specific de novo questions | national health portal, health-related applications | 6 |
| Smola et al (2024) [48] | Poland | Polish adult Internet users aged 18–75 (n=1661) | Polish version of the Transactional e-Health Literacy Instrument | Items asking about the use of e-health services in the preceding 12 months | IPAP, health info portals, lab results websites, paid advice portals, e-prescription websites | 8 |
| Khan et al (2018) [49] | Denmark | Cardiac surgery patients (n=33) | eHEALS | Questionnaire on use of Internet for health information | [Activeheart.dk](https://activeheart.dk/) portal, general Internet search | 4 |
| Lotto et al (2023) [50] | Brazil | Undergraduate students aged 15–55 years from two universities in São Paulo and Santa Catarina (n=521) | Brazilian Portuguese version of eHEALS | items on health information seeking behavior in the last 24 hours (yes/no); consumption of health information in newspapers, radio or TV, social media, and websites | Social media, newspapers/radio/TV, websites | 6 |
| Gazibara et al (2025) [51] | Serbia | High school students aged 14–19 years from four high schools in Belgrade (n=702) | Serbian version of eHEALS | whether students used the Internet to seek information about mental health (yes/no); browsing frequency across different online platforms | Health forums, health blogs, social media, websites run by physicians, websites run by health institutions, health portals, YouTube | 7 |

^a^ eHEALS: eHealth Literacy Scale.

^b^ HINTS: Health Information National Trends Survey.

References:

33. Mitsutake S, Oka K, Okan O, et al. eHealth literacy and web-based health information-seeking behaviors on COVID-19 in Japan: internet-based mixed methods study. J Med Internet Res. Jul 11, 2024;26:e57842. [doi: 10.2196/57842] [Medline: 38990625]

34. Liu D, Yang S, Cheng CY, Cai L, Su J. Online health information seeking, eHealth literacy, and health behaviors among Chinese internet users: cross-sectional survey study. J Med Internet Res. Oct 18, 2024;26:e54135. [doi: 10.2196/54135] [Medline: 39423374]

35. Yun HS, Bickmore T. Online health information-seeking in the era of large language models: cross-sectional web-based survey study. J Med Internet Res. Mar 31, 2025;27:e68560. [doi: 10.2196/68560] [Medline: 40163112]

36. Tian H, Chen J. Associations among online health information seeking behaviors, electronic health literacy and food neophobia: a cross-sectional study. Inquiry. 2023;60:469580231217982. [doi: 10.1177/00469580231217982] [Medline: 38018557]

37. Gazibara T, Cakic M, Cakic J, Grgurevic A, Pekmezovic T. Patterns of online health information seeking after visiting a physician: perceptions of adolescents from high schools in central Belgrade, Serbia. Fam Pract. Jun 17, 2021;38(3):231-237. [doi: 10.1093/fampra/cmaa118] [Medline: 33096547]

38. Chen J, Tian H. Associations and gender differences between OHI-seeking behaviors and eHealth literacy among Chinese university students. Rev Esc Enferm USP. 2024;58:e20230340. [doi: 10.1590/1980-220X-REEUSP-2023-0340en] [Medline: 38602487]

39. Shi G, Yu J, Zhang J, Zhao J, Peng Z, Shang L. Factors affecting online health information-seeking behavior in young and middle-aged patients with stroke. PLoS One. 2025;20(4):e0321791. [doi: 10.1371/journal.pone.0321791] [Medline: 40294006]

40. Islam MM, Touray M, Yang HC, et al. e-Health literacy and health information seeking behavior among university students in Bangladesh. Stud Health Technol Inform. 2017;245:122-125. [doi: 10.3233/978-1-61499-830-3-122] [Medline: 29295065]

41. Tennant B, Stellefson M, Dodd V, et al. eHealth literacy and web 2.0 health information seeking behaviors among baby boomers and older adults. J Med Internet Res. Mar 17, 2015;17(3):e70. [doi: 10.2196/jmir.3992] [Medline: 25783036]

42. Wong DKK, Cheung MK. Online health information seeking and eHealth literacy among patients attending a primary care clinic in Hong Kong: a cross-sectional survey. J Med Internet Res. Mar 27, 2019;21(3):e10831. [doi: 10.2196/10831] [Medline: 30916666]

43. James DCS, Harville C II. eHealth literacy, online help-seeking behavior, and willingness to participate in mHealth chronic disease research among African Americans, Florida, 2014-2015. Prev Chronic Dis. Nov 17, 2016;13:E156. [doi: 10.5888/pcd13.160210] [Medline: 27854421]

44. Quinn S, Bond R, Nugent C. Quantifying health literacy and eHealth literacy using existing instruments and browser-based software for tracking online health information seeking behavior. Comput Human Behav. Apr 2017;69:256-267. [doi: 10.1016/j.chb.2016.12.032]

45. Saffarzadeh A. Reconceptualizing health literacy and the eHealth Literacy Scale (eHEALS): evaluation of psychometric properties, subdimensions, and health-related internet searching behavior in adult outpatients visiting a tertiary care clinic. University of California; 2015. URL: <https://escholarship.org/uc/item/71x482hb> [Accessed 2026-06-24]

46. Chang FC, Chiu CH, Chen PH, et al. Relationship between parental and adolescent eHealth literacy and online health information seeking in Taiwan. Cyberpsychol Behav Soc Netw. Oct 2015;18(10):618-624. [doi: 10.1089/cyber.2015.0110] [Medline: 26375050]

47. Kyaw MY, Aung MN, Koyanagi Y, et al. Sociodigital determinants of eHealth literacy and related impact on health outcomes and eHealth use in Korean older adults: community-based cross-sectional survey. JMIR Aging. Aug 13, 2024;7:e56061. [doi: 10.2196/56061] [Medline: 39140239]

48. Smoła P, Zwierczyk U, Duplaga M. Transactional e-health literacy and its association with e-health services use in Polish adults: a cross-sectional study. Front Digit Health. 2024;6:1458650. [doi: 10.3389/fdgth.2024.1458650] [Medline: 39650753]

49. Khan D, Fjerbæk A, Andreasen JJ, Thorup CB, Dinesen B. Cardiac surgery patients’ e-health literacy and their use of a digital portal. Health Educ J. Jun 2018;77(4):482-494. [doi: 10.1177/0017896918756435]

50. Lotto M, Maschio KF, Silva KK, Ayala Aguirre PE, Cruvinel A, Cruvinel T. eHEALS as a predictive factor of digital health information seeking behavior among Brazilian undergraduate students. Health Promot Int. Aug 1, 2023;38(4):daab182. [doi: 10.1093/heapro/daab182] [Medline: 34718563]

51. Gazibara T, Cakic J, Cakic M, Grgurevic A, Pekmezovic T. Factors associated with online information seeking about mental health among high school students in Belgrade, Serbia. Camb prisms Glob ment health. 2025;12:e94. [doi: 10.1017/gmh.2025.10026]

52. Maitz E, Maitz K, Sendlhofer G, et al. Internet-based health information-seeking behavior of students aged 12 to 14 years: mixed methods study. J Med Internet Res. May 26, 2020;22(5):e16281. [doi: 10.2196/16281] [Medline: 32209532]

53. Pho AT, Bakken S, Lunn MR, et al. Online health information seeking, health literacy, and human papillomavirus vaccination among transgender and gender-diverse people. J Am Med Inform Assoc. Jan 12, 2022;29(2):285-295. [doi: 10.1093/jamia/ocab150] [Medline: 34383916]

54. Jiao W, Chang A, Ho M, Lu Q, Liu MT, Schulz PJ. Predicting and empowering health for generation Z by comparing health information seeking and digital health literacy: cross-sectional questionnaire study. J Med Internet Res. Oct 30, 2023;25:e47595. [doi: 10.2196/47595] [Medline: 37902832]

55. Robinson-Whelen S, Hughes RB, Alhusen JL, Beers L, Minard CG, Davidson D. Health information seeking in the digital age: a national survey of women with disabilities. Disabil Rehabil. Aug 2023;45(17):2751-2760. [doi: 10.1080/09638288.2022.2105960] [Medline: 35916449]

56. KHademian F, Arshadi Montazer MR, Aslani A. Web-based health information seeking and eHealth literacy among college students. A self-report study. Invest Educ Enferm. Feb 2020;38(1):e08. [doi: 10.17533/udea.iee.v38n1e08] [Medline: 32124576]

57. Htet H, Wichaidit W, Sriplung H, et al. Do electronic health literacy and online health information-seeking behavior mediate the effects of socio-demographic factors on COVID-19- and non-communicable disease-related behaviors among Myanmar migrants in Southern Thailand? Cureus. Nov 2023;15(11):e49090. [doi: 10.7759/cureus.49090] [Medline: 38125220]

58. Kim S, Park K, Jo HS. Gap between perceived eHealth literacy and ability to use online cancer-related information. J Korean Med Sci. Jun 22, 2020;35(24):e187. [doi: 10.3346/jkms.2020.35.e187]

59. Lee K, Hoti K, Hughes JD, Emmerton LM. Consumer use of “Dr Google”: a survey on health information-seeking behaviors and navigational needs. J Med Internet Res. Dec 29, 2015;17(12):e288. [doi: 10.2196/jmir.4345] [Medline: 26715363]

60. Stellefson ML, Shuster JJ, Chaney BH, et al. Web-based health information seeking and eHealth literacy among patients living with chronic obstructive pulmonary disease (COPD). Health Commun. Dec 2018;33(12):1410-1424. [doi: 10.1080/10410236.2017.1353868] [Medline: 28872905]

61. Peimani M, Stewart AL, Ghodssi-Ghassemabadi R, Nasli-Esfahani E, Ostovar A. The moderating role of e-health literacy and patient-physician communication in the relationship between online diabetes information-seeking behavior and self-care practices among individuals with type 2 diabetes. BMC Prim Care. Dec 30, 2024;25(1):442. [doi: 10.1186/s12875-024-02695-9] [Medline: 39736551]

62. Xie B. Older adults, e-health literacy, and collaborative learning: an experimental study. J Am Soc Inf Sci. May 2011;62(5):933-946. [doi: 10.1002/asi.21507]

63. Puspita N, Kurniawan AH, Tias CAN. Exploring the relationship between e-health literacy and online health information-seeking behaviour among pharmacy students in Indonesia. Pharm Educ. 2024;24(1):304-310. [doi: 10.46542/pe.2024.241.304310]

64. Ramstad KJ, Brørs G, Pettersen TR, et al. eHealth technology use and eHealth literacy after percutaneous coronary intervention. Eur J Cardiovasc Nurs. Jul 19, 2023;22(5):472-481. [doi: 10.1093/eurjcn/zvac087] [Medline: 36190843]
