# Supplementary material for: The Relation Between eHealth Literacy and Online Health Information–Seeking Behavior: Systematic Review and Meta-Analysis
Source: J Med Internet Res. 2026 Jul 15;28:e93578. doi: 10.2196/93578 (PMC13372218; doi:10.2196/93578)
Supplement: Multimedia Appendix 3 [file jmir-v28-e93578-s003.docx]

Multimedia Appendix 3. Risk of bias assessment for the included studies using the modified Newcastle-Ottawa Scale.

| **Study (Year)** | **Selection** | | | | | **Comparability** | **Outcome** | | | **Total** |
| --- | --- | --- | --- | --- | --- | --- | --- | --- | --- | --- |
|  | **Representative-ness of the samples ^a^** | **Sample size calculation ^b^** | **Non-respondents ^c^** | | **Use of validated eHealth literacy measurement tool ^d^** | **Adjustment of confounding factors ^e^** | **Use of validated instruments for outcome measurement ^f^** | **Ascertainment of the outcome ^g^** | **Statistical test ^h^** |  |
| Mitsutake S et al (2024) [33] | ★ |  | |  | ★ | ★★ |  | ★ | ★ | 6 |
| Maitz et al (2020) [52] |  |  | | ★ | ★ |  |  | ★ | ★ | 4 |
| Pho et al (2022) [53] |  |  | |  | ★ | ★★ |  | ★ | ★ | 5 |
| Liu et al (2024) [34] |  | ★ | |  |  | ★★ |  | ★ | ★ | 5 |
| Yun and Bickmore (2025) [35] |  | ★ | | ★ | ★ |  |  | ★ | ★ | 5 |
| Jiao et al (2023) [54] |  | ★ | | ★ | ★ |  |  | ★ | ★ | 5 |
| Robinson-Whelen et al(2023) [55] |  |  | | ★ | ★ |  |  | ★ | ★ | 4 |
| Kademian et al (2020) [56] |  |  | | ★ |  |  |  | ★ | ★ | 3 |
| Tian and Chen (2023) [36] |  |  | | ★ | ★ |  | ★ | ★ | ★ | 5 |
| Htet et al (2023) [57] | ★ | ★ | |  | ★ |  |  | ★ | ★ | 5 |
| Kim et al. (2020) [58] |  |  | |  | ★ |  |  | ★ | ★ | 3 |
| Gazibara et al(2021) [37] | ★ | ★ | |  | ★ | ★★ |  | ★ | ★ | 7 |
| Chen and Tian (2024) [38] |  |  | | ★ | ★ |  | ★ | ★ | ★ | 5 |
| Shi G et al (2025) [39] |  | ★ | | ★ | ★ | ★★ |  | ★ | ★ | 7 |
| Lee K et al (2015) [59] |  | ★ | |  | ★ | ★★ |  | ★ | ★ | 6 |
| Islam et al(2017) [40] |  |  | |  | ★ | ★ |  | ★ | ★ | 4 |
| Tennant B et al (2015) [41] | ★ |  | |  | ★ | ★★ | ★ | ★ | ★ | 7 |
| Stellefson M et al(2018) [60] | ★ |  | |  | ★ | ★★ | ★ | ★ | ★ | 7 |
| Wong and Cheung (2019) [42] | ★ | ★ | | ★ | ★ | ★★ | ★ | ★ | ★ | 9 |
| James and Harville (2016) [43] |  |  | |  | ★ |  | ★ | ★ | ★ | 4 |
| Pelmani et al (2024) [61] | ★ | ★ | |  | ★ | ★★ | ★ | ★ | ★ | 8 |
| Bo Xie (2011) [62] |  |  | |  | ★ | ★★ | ★ | ★ | ★ | 6 |
| Quinn S et al(2017) [44] |  |  | |  | ★ | ★ | ★ | ★ | ★ | 5 |
| Puspita N et al (2024) [63] |  | ★ | |  | ★ |  | ★ | ★ | ★ | 5 |
| Saffarzadeh A (2015) [45] |  |  | | ★ | ★ | ★★ | ★ | ★ | ★ | 7 |
| Chang et al. (2015) [46] | ★ |  | |  | ★ | ★★ | ★ | ★ | ★ | 7 |
| Kyaw et al. (2024) [47] |  | ★ | | ★ | ★ | ★★ | ★ | ★ | ★ | 8 |
| Ramstad K.J. et al. (2023) [64] |  |  | | ★ | ★ | ★★ |  | ★ | ★ | 6 |
| Smola P et al. (2024) [48] | ★ | ★ | |  | ★ | ★★ | ★ | ★ | ★ | 8 |
| Khan D et al. (2018) [49] |  |  | |  | ★ |  | ★ | ★ | ★ | 4 |
| Lotto et al. (2023) [50] |  | ★ | |  | ★ | ★★ |  | ★ | ★ | 6 |
| Gazibara et al. (2025) [51] | ★ | ★ | |  | ★ | ★★ |  | ★ | ★ | 7 |

^a^ Description of the sampling strategy (no star);

^b^ Performed (one star), Not performed (no star);

^c^ Losses ≤20% (one star), Losses >20% or not reported (no star);

^d^ Yes (one star), No (no star);

^e^ Adjusted by one confounding factor (one star), Adjusted by other confounding factors (one star), Did not control confounding factors (no star), (Maximum two stars);

^f^ Yes (one star), No (no star);

^g^ Primary data (one star), Secondary data or not reported (no star);

^h^ Appropriate statistical test and clearly described including confidence intervals or the probability level (one star), Inappropriate, incomplete, or not described statistical test (no star).

References:

33. Mitsutake S, Oka K, Okan O, et al. eHealth literacy and web-based health information-seeking behaviors on COVID-19 in Japan: internet-based mixed methods study. J Med Internet Res. Jul 11, 2024;26:e57842. [doi: 10.2196/57842] [Medline: 38990625]

34. Liu D, Yang S, Cheng CY, Cai L, Su J. Online health information seeking, eHealth literacy, and health behaviors among Chinese internet users: cross-sectional survey study. J Med Internet Res. Oct 18, 2024;26:e54135. [doi: 10.2196/54135] [Medline: 39423374]

35. Yun HS, Bickmore T. Online health information-seeking in the era of large language models: cross-sectional web-based survey study. J Med Internet Res. Mar 31, 2025;27:e68560. [doi: 10.2196/68560] [Medline: 40163112]

36. Tian H, Chen J. Associations among online health information seeking behaviors, electronic health literacy and food neophobia: a cross-sectional study. Inquiry. 2023;60:469580231217982. [doi: 10.1177/00469580231217982] [Medline: 38018557]

37. Gazibara T, Cakic M, Cakic J, Grgurevic A, Pekmezovic T. Patterns of online health information seeking after visiting a physician: perceptions of adolescents from high schools in central Belgrade, Serbia. Fam Pract. Jun 17, 2021;38(3):231-237. [doi: 10.1093/fampra/cmaa118] [Medline: 33096547]

38. Chen J, Tian H. Associations and gender differences between OHI-seeking behaviors and eHealth literacy among Chinese university students. Rev Esc Enferm USP. 2024;58:e20230340. [doi: 10.1590/1980-220X-REEUSP-2023-0340en] [Medline: 38602487]

39. Shi G, Yu J, Zhang J, Zhao J, Peng Z, Shang L. Factors affecting online health information-seeking behavior in young and middle-aged patients with stroke. PLoS One. 2025;20(4):e0321791. [doi: 10.1371/journal.pone.0321791] [Medline: 40294006]

40. Islam MM, Touray M, Yang HC, et al. e-Health literacy and health information seeking behavior among university students in Bangladesh. Stud Health Technol Inform. 2017;245:122-125. [doi: 10.3233/978-1-61499-830-3-122] [Medline: 29295065]

41. Tennant B, Stellefson M, Dodd V, et al. eHealth literacy and web 2.0 health information seeking behaviors among baby boomers and older adults. J Med Internet Res. Mar 17, 2015;17(3):e70. [doi: 10.2196/jmir.3992] [Medline: 25783036]

42. Wong DKK, Cheung MK. Online health information seeking and eHealth literacy among patients attending a primary care clinic in Hong Kong: a cross-sectional survey. J Med Internet Res. Mar 27, 2019;21(3):e10831. [doi: 10.2196/10831] [Medline: 30916666]

43. James DCS, Harville C II. eHealth literacy, online help-seeking behavior, and willingness to participate in mHealth chronic disease research among African Americans, Florida, 2014-2015. Prev Chronic Dis. Nov 17, 2016;13:E156. [doi: 10.5888/pcd13.160210] [Medline: 27854421]

44. Quinn S, Bond R, Nugent C. Quantifying health literacy and eHealth literacy using existing instruments and browser-based software for tracking online health information seeking behavior. Comput Human Behav. Apr 2017;69:256-267. [doi: 10.1016/j.chb.2016.12.032]

45. Saffarzadeh A. Reconceptualizing health literacy and the eHealth Literacy Scale (eHEALS): evaluation of psychometric properties, subdimensions, and health-related internet searching behavior in adult outpatients visiting a tertiary care clinic. University of California; 2015. URL: https://escholarship.org/uc/item/71x482hb [Accessed 2026-06-24]

46. Chang FC, Chiu CH, Chen PH, et al. Relationship between parental and adolescent eHealth literacy and online health information seeking in Taiwan. Cyberpsychol Behav Soc Netw. Oct 2015;18(10):618-624. [doi: 10.1089/cyber.2015.0110] [Medline: 26375050]

47. Kyaw MY, Aung MN, Koyanagi Y, et al. Sociodigital determinants of eHealth literacy and related impact on health outcomes and eHealth use in Korean older adults: community-based cross-sectional survey. JMIR Aging. Aug 13, 2024;7:e56061. [doi: 10.2196/56061] [Medline: 39140239]

48. Smoła P, Zwierczyk U, Duplaga M. Transactional e-health literacy and its association with e-health services use in Polish adults: a cross-sectional study. Front Digit Health. 2024;6:1458650. [doi: 10.3389/fdgth.2024.1458650] [Medline: 39650753]

49. Khan D, Fjerbæk A, Andreasen JJ, Thorup CB, Dinesen B. Cardiac surgery patients’ e-health literacy and their use of a digital portal. Health Educ J. Jun 2018;77(4):482-494. [doi: 10.1177/0017896918756435]

50. Lotto M, Maschio KF, Silva KK, Ayala Aguirre PE, Cruvinel A, Cruvinel T. eHEALS as a predictive factor of digital health information seeking behavior among Brazilian undergraduate students. Health Promot Int. Aug 1, 2023;38(4):daab182. [doi: 10.1093/heapro/daab182] [Medline: 34718563]

51. Gazibara T, Cakic J, Cakic M, Grgurevic A, Pekmezovic T. Factors associated with online information seeking about mental health among high school students in Belgrade, Serbia. Camb prisms Glob ment health. 2025;12:e94. [doi: 10.1017/gmh.2025.10026]

52. Maitz E, Maitz K, Sendlhofer G, et al. Internet-based health information-seeking behavior of students aged 12 to 14 years: mixed methods study. J Med Internet Res. May 26, 2020;22(5):e16281. [doi: 10.2196/16281] [Medline: 32209532]

53. Pho AT, Bakken S, Lunn MR, et al. Online health information seeking, health literacy, and human papillomavirus vaccination among transgender and gender-diverse people. J Am Med Inform Assoc. Jan 12, 2022;29(2):285-295. [doi: 10.1093/jamia/ocab150] [Medline: 34383916]

54. Jiao W, Chang A, Ho M, Lu Q, Liu MT, Schulz PJ. Predicting and empowering health for generation Z by comparing health information seeking and digital health literacy: cross-sectional questionnaire study. J Med Internet Res. Oct 30, 2023;25:e47595. [doi: 10.2196/47595] [Medline: 37902832]

55. Robinson-Whelen S, Hughes RB, Alhusen JL, Beers L, Minard CG, Davidson D. Health information seeking in the digital age: a national survey of women with disabilities. Disabil Rehabil. Aug 2023;45(17):2751-2760. [doi: 10.1080/09638288.2022.2105960] [Medline: 35916449]

56. KHademian F, Arshadi Montazer MR, Aslani A. Web-based health information seeking and eHealth literacy among college students. A self-report study. Invest Educ Enferm. Feb 2020;38(1):e08. [doi: 10.17533/udea.iee.v38n1e08] [Medline: 32124576]

57. Htet H, Wichaidit W, Sriplung H, et al. Do electronic health literacy and online health information-seeking behavior mediate the effects of socio-demographic factors on COVID-19- and non-communicable disease-related behaviors among Myanmar migrants in Southern Thailand? Cureus. Nov 2023;15(11):e49090. [doi: 10.7759/cureus.49090] [Medline: 38125220]

58. Kim S, Park K, Jo HS. Gap between perceived eHealth literacy and ability to use online cancer-related information. J Korean Med Sci. Jun 22, 2020;35(24):e187. [doi: 10.3346/jkms.2020.35.e187]

59. Lee K, Hoti K, Hughes JD, Emmerton LM. Consumer use of “Dr Google”: a survey on health information-seeking behaviors and navigational needs. J Med Internet Res. Dec 29, 2015;17(12):e288. [doi: 10.2196/jmir.4345] [Medline: 26715363]

60. Stellefson ML, Shuster JJ, Chaney BH, et al. Web-based health information seeking and eHealth literacy among patients living with chronic obstructive pulmonary disease (COPD). Health Commun. Dec 2018;33(12):1410-1424. [doi: 10.1080/10410236.2017.1353868] [Medline: 28872905]

61. Peimani M, Stewart AL, Ghodssi-Ghassemabadi R, Nasli-Esfahani E, Ostovar A. The moderating role of e-health literacy and patient-physician communication in the relationship between online diabetes information-seeking behavior and self-care practices among individuals with type 2 diabetes. BMC Prim Care. Dec 30, 2024;25(1):442. [doi: 10.1186/s12875-024-02695-9] [Medline: 39736551]

62. Xie B. Older adults, e-health literacy, and collaborative learning: an experimental study. J Am Soc Inf Sci. May 2011;62(5):933-946. [doi: 10.1002/asi.21507]

63. Puspita N, Kurniawan AH, Tias CAN. Exploring the relationship between e-health literacy and online health information-seeking behaviour among pharmacy students in Indonesia. Pharm Educ. 2024;24(1):304-310. [doi: 10.46542/pe.2024.241.304310]

64. Ramstad KJ, Brørs G, Pettersen TR, et al. eHealth technology use and eHealth literacy after percutaneous coronary intervention. Eur J Cardiovasc Nurs. Jul 19, 2023;22(5):472-481. [doi: 10.1093/eurjcn/zvac087] [Medline: 36190843]
