# Supplementary material for: The Relation Between eHealth Literacy and Online Health Information–Seeking Behavior: Systematic Review and Meta-Analysis
Source: J Med Internet Res. 2026 Jul 15;28:e93578. doi: 10.2196/93578 (PMC13372218; doi:10.2196/93578)
Supplement: Multimedia Appendix 4 [file jmir-v28-e93578-s004.docx]

**Multimedia Appendix 4.** The relationship between eHealth literacy and online health information seeking behaviors in the included studies.

| **Study (Year)** | **eHealth literacy instrument** | **Types of online health information seeking behaviors** | **Relationship** |
| --- | --- | --- | --- |
| Mitsutake S et al (2024) [33] | Japanese version of eHealth Literacy Scale (eHEALS) | Searching for information on preventive measures, vaccination, infection status, symptoms, routes of infection | Higher eHealth literacy（eHL） significantly positively associated with preventive information seeking; also linked to diagnosis/treatment and self-management seeking. |
| Maitz et al (2020) [52] | eHEALS | Searching for health information online, including evaluating reliability, learning about medical procedures, and applying information in decision-making | eHEALS score positively correlated with self-efficacy; students overestimated their eHL; short workshop slightly improved scores and awareness. |
| Pho et al (2022) [53] | eHEALS | Online search for general health information, vaccine-related information, and use of social networking sites for health purposes | Participants had higher odds of Human Papillomavirus vaccination overall, but lower odds after searching online for vaccine information; no moderating effect from general or eHL. |
| Liu et al (2024) [34] | Adapted eHEALS | Looking for information on healthy lifestyle, disease prevention, diagnosis and treatment options, and self-management of chronic conditions | eHL (both **functional** and **critical** literacy) was **significantly and positively associated** with online health information seeking. |
| Yun and Bickmore (2025) [35] | eHEALS | Participants searched for information on **health conditions or symptoms, medications, medical procedures or treatments, diet, fitness, and self-diagnosis** | **eHL** showed a **positive correlation** with the number of source types used , following information, and cross-checking information . |
| Jiao et al (2023) [54] | eHEALS | Not specified by content (the survey measured only sources, not topic categories) | Digital health literacy was significantly and positively correlated with web-based information-seeking behavior. |
| Robinson-Whelen et al (2023) [55] | eHEALS | menstruation; fertility; contraception; pre-pregnancy/family planning; pregnancy, labor and delivery; bladder/bowel problems; pelvic-floor problems; menopause; women’s cancers; cancer screening; well-woman exams; finding a physician; insurance coverage | Although participants reported high eHL, confidence in eHL skills did not eliminate challenges in online information seeking. |
| Kademian et al (2020) [56] | Adapted eHEALS | Disease symptoms, physical illness, treatments, diagnosis; less on prevention, mental health, emotional support | Higher levels of eHL were associated with more active health information-seeking behaviors. |
| Tian and Chen (2023) [36] | Chinese version of eHEALS | diet; physical exercise; health/medical information; hospitals or doctors; social media sharing (reading, writing); purchasing health products; smoking cessation; drinking cessation; online appointments; disease-specific internet communities | eHL was positively correlated with preventive information–seeking behaviors. |
| Htet et al (2023) [57] | eHEALS | COVID-19 protective behavior and vaccination；Non-communicable disease risk behaviors, including smoking, betel chewing, alcohol consumption, and substance abuse | The study found that eHL literacy and online health information seeking did not have significant mediating roles. |
| Kim et al (2020) [58] | Korean version of eHEALS | Cancer treatment–related information | eHL was significantly and positively correlated with diagnosis and treatment–related information seeking behavior. |
| Gazibara et al (2021) [37] | Serbian version of eHEALS | Illicit drugs; Sexually transmitted infections; Medications; Cancer; Mental health problems | The self-reported eHL literacy significantly increased with the perceived frequency of online health information seeking. |
| Chen and Tian (2024) [38] | Chinese version of eHEALS | Diet and nutrition, physical exercise, smoking cessation, drinking cessation, health information sharing via social media, hospital/doctor information, medical information, health product purchasing, online health reservation, specific disease community participation | eHL was significantly positively correlated with preventive information seeking behavior. |
| Shi G et al (2025) [39] | eHEALS | Information on hospitals/doctors, physical exercise, smoking cessation, health/medical information, drinking cessation, nutrition/diet, health product purchasing, online health reservation, specific disease communities. | eHL was significantly and positively correlated with online Health Information seeking behaviors (OHIS) . |
| Lee K et al (2015) [59] | eHEALS | Seeking information to be more informed about health, managing own chronic health conditions, clarifying information from health professionals, checking information discussed during consultations with health professionals | eHL and OHIS have a clear negative associative relationship. |
| Islam et al (2017) [40] | eHEALS | Use of social media, web-based support groups, and blogs for health information. | eHL was positively associated with the use of Web 2.0 for health information seeking and sharing. |
| Tennant B et al. (2015) [41] | eHEALS | Use of social networking sites, web-based support groups, and blogs for locating or sharing health information. | Higher eHL was significantly associated with Web 2.0 use for health information. |
| Stellefson M et al. (2018) [60] | eHEALS | Seeking and sharing Chronic Obstructive Pulmonary Disease–related health information via electronic devices | eHL was significantly and positively correlated with most types of OHIS except for nutrition information seeking and online health product purchasing. |
| Wong and Cheung (2019) [42] | eHEALS | Seeking online health information (e.g., symptoms, diseases, medications, healthy behaviors) via devices | Higher eHEALS scores were significant predictors of more online health information seeking. |
| James and Harville (2016) [43] | eHEALS | Searching for information on health and wellness, nutrition/dieting, medication use, diabetes, stress/anxiety/depression, children’s health, heart disease, cancer, sexually transmitted infections, tobacco/alcohol/drugs, asthma, and HIV/AIDS | eHL was significantly higher among those who sought online health information and among those willing to participate in Mobile health research. |
| Pelmani et al (2024) [61] | Persian version of eHEALS | Participating in online support groups, using email or the internet to communicate with physicians, and using the internet to look up diabetes-related information | eHL was weakly positively correlated with online diabetes information-seeking behavior. |
| Bo Xie (2011) [62] | eHEALS | Using the NIHSeniorHealth.gov and MedlinePlus.gov websites to find health information | eHL moderated the positive association between online diabetes information-seeking. |
| Quinn S et al (2017) [44] | eHEALS | Diabetes, obesity, influenza, nutrition, analgesic medication | eHL was not significantly correlated with health question scores;eHEALS is weakly negatively correlated with the search difficulty score. |
| Puspita N et al (2024) [63] | Indonesian version of eHEALS | Health issues, assignment references, updating health information | eHL was significantly and positively associated with the frequency of online health information seeking behavior. |
| Saffarzadeh A (2015) [45] | eHEALS | Disease-related information, physician quality information | Lower eHL was significantly associated with decreased use of high-quality health websites and less frequent searching for physician-related information online. |
| Chang et al (2015) [46] | eHEALS | General health information | Higher adolescent eHL and higher parental eHL were both significantly associated with increased adolescent online health information seeking. |
| Kyaw et al (2024) [47] | eHEALS | Diet, exercise, health care services, long-term care services | Higher eHL was significantly associated with more frequent use of digital technology for health-related purposes. |
| Ramstad K.J. et al (2023) [64] | eHEALS | Health, prevention, illness, treatment, and use of health applications | Higher eHL was significantly associated with increased use of the internet for health information and health-related apps. |
| Smola P et al (2024) [48] | Polish version of the Transactional eHL Literacy Instrument | Remote physician advice, Internet Patient Account Portal, general health information portals, laboratory test results websites, paid medical advice portals, and e-prescription services | Functional and Translational eHL positively associated with using health information portals, the Internet Patient Account Portal and lab result websites; Communicative eHL positively associated with paid medical advice portals but negatively with health information portals; Critical eHL positively associated with paid medical advice portals and e-prescription websites but negatively with remote physician advice and lab result websites. |
| Khan D et al (2018) [49] | eHEALS | Use of the Activeheart.dk digital portal for postoperative information, exercises, lifestyle changes, and coping strategies | No significant relationship was found between patients' self-reported eHL literacy skills and their actual use of the digital portal. |
| Lotto et al (2023) [50] | Brazilian Portuguese version of eHEALS | Health information seeking in the last 24 hours; consumption of health information in newspapers, radio or TV, social media, and websites | Higher eHEALS scores (>28) significantly predicted recent health information seeking behavior (OR = 1.57, 95% CI 1.03–2.39, p = 0.035). The eHEALS demonstrated adequate predictive validity for digital health information seeking among Brazilian undergraduate students. |
| Gazibara et al (2025) [51] | Serbian version of eHEALS | Online information seeking about mental health (depressive symptoms, anxiety, other mood-related problems, anger, attention deficit, eating disorders) | Lower eHEALS score was independently associated with online information seeking about mental health (adjusted OR = 0.95, 95% CI 0.93–0.98, p = 0.001). Students who sought mental health information online had significantly lower eHEALS scores (median 25 vs 26, p = 0.016), suggesting an inverse association between eHL and mental health–specific OHIS. |

eHealth Literacy Scale (eHEALS)
eHealth literacy（eHL）
Online Health Information seeking behaviors (OHIS)

References:

33. Mitsutake S, Oka K, Okan O, et al. eHealth literacy and web-based health information-seeking behaviors on COVID-19 in Japan: internet-based mixed methods study. J Med Internet Res. Jul 11, 2024;26:e57842. [doi: 10.2196/57842] [Medline: 38990625]

34. Liu D, Yang S, Cheng CY, Cai L, Su J. Online health information seeking, eHealth literacy, and health behaviors among Chinese internet users: cross-sectional survey study. J Med Internet Res. Oct 18, 2024;26:e54135. [doi: 10.2196/54135] [Medline: 39423374]

35. Yun HS, Bickmore T. Online health information-seeking in the era of large language models: cross-sectional web-based survey study. J Med Internet Res. Mar 31, 2025;27:e68560. [doi: 10.2196/68560] [Medline: 40163112]

36. Tian H, Chen J. Associations among online health information seeking behaviors, electronic health literacy and food neophobia: a cross-sectional study. Inquiry. 2023;60:469580231217982. [doi: 10.1177/00469580231217982] [Medline: 38018557]

37. Gazibara T, Cakic M, Cakic J, Grgurevic A, Pekmezovic T. Patterns of online health information seeking after visiting a physician: perceptions of adolescents from high schools in central Belgrade, Serbia. Fam Pract. Jun 17, 2021;38(3):231-237. [doi: 10.1093/fampra/cmaa118] [Medline: 33096547]

38. Chen J, Tian H. Associations and gender differences between OHI-seeking behaviors and eHealth literacy among Chinese university students. Rev Esc Enferm USP. 2024;58:e20230340. [doi: 10.1590/1980-220X-REEUSP-2023-0340en] [Medline: 38602487]

39. Shi G, Yu J, Zhang J, Zhao J, Peng Z, Shang L. Factors affecting online health information-seeking behavior in young and middle-aged patients with stroke. PLoS One. 2025;20(4):e0321791. [doi: 10.1371/journal.pone.0321791] [Medline: 40294006]

40. Islam MM, Touray M, Yang HC, et al. e-Health literacy and health information seeking behavior among university students in Bangladesh. Stud Health Technol Inform. 2017;245:122-125. [doi: 10.3233/978-1-61499-830-3-122] [Medline: 29295065]

41. Tennant B, Stellefson M, Dodd V, et al. eHealth literacy and web 2.0 health information seeking behaviors among baby boomers and older adults. J Med Internet Res. Mar 17, 2015;17(3):e70. [doi: 10.2196/jmir.3992] [Medline: 25783036]

42. Wong DKK, Cheung MK. Online health information seeking and eHealth literacy among patients attending a primary care clinic in Hong Kong: a cross-sectional survey. J Med Internet Res. Mar 27, 2019;21(3):e10831. [doi: 10.2196/10831] [Medline: 30916666]

43. James DCS, Harville C II. eHealth literacy, online help-seeking behavior, and willingness to participate in mHealth chronic disease research among African Americans, Florida, 2014-2015. Prev Chronic Dis. Nov 17, 2016;13:E156. [doi: 10.5888/pcd13.160210] [Medline: 27854421]

44. Quinn S, Bond R, Nugent C. Quantifying health literacy and eHealth literacy using existing instruments and browser-based software for tracking online health information seeking behavior. Comput Human Behav. Apr 2017;69:256-267. [doi: 10.1016/j.chb.2016.12.032]

45. Saffarzadeh A. Reconceptualizing health literacy and the eHealth Literacy Scale (eHEALS): evaluation of psychometric properties, subdimensions, and health-related internet searching behavior in adult outpatients visiting a tertiary care clinic. University of California; 2015. URL: https://escholarship.org/uc/item/71x482hb [Accessed 2026-06-24]

46. Chang FC, Chiu CH, Chen PH, et al. Relationship between parental and adolescent eHealth literacy and online health information seeking in Taiwan. Cyberpsychol Behav Soc Netw. Oct 2015;18(10):618-624. [doi: 10.1089/cyber.2015.0110] [Medline: 26375050]

47. Kyaw MY, Aung MN, Koyanagi Y, et al. Sociodigital determinants of eHealth literacy and related impact on health outcomes and eHealth use in Korean older adults: community-based cross-sectional survey. JMIR Aging. Aug 13, 2024;7:e56061. [doi: 10.2196/56061] [Medline: 39140239]

48. Smoła P, Zwierczyk U, Duplaga M. Transactional e-health literacy and its association with e-health services use in Polish adults: a cross-sectional study. Front Digit Health. 2024;6:1458650. [doi: 10.3389/fdgth.2024.1458650] [Medline: 39650753]

49. Khan D, Fjerbæk A, Andreasen JJ, Thorup CB, Dinesen B. Cardiac surgery patients’ e-health literacy and their use of a digital portal. Health Educ J. Jun 2018;77(4):482-494. [doi: 10.1177/0017896918756435]

50. Lotto M, Maschio KF, Silva KK, Ayala Aguirre PE, Cruvinel A, Cruvinel T. eHEALS as a predictive factor of digital health information seeking behavior among Brazilian undergraduate students. Health Promot Int. Aug 1, 2023;38(4):daab182. [doi: 10.1093/heapro/daab182] [Medline: 34718563]

51. Gazibara T, Cakic J, Cakic M, Grgurevic A, Pekmezovic T. Factors associated with online information seeking about mental health among high school students in Belgrade, Serbia. Camb prisms Glob ment health. 2025;12:e94. [doi: 10.1017/gmh.2025.10026]

52. Maitz E, Maitz K, Sendlhofer G, et al. Internet-based health information-seeking behavior of students aged 12 to 14 years: mixed methods study. J Med Internet Res. May 26, 2020;22(5):e16281. [doi: 10.2196/16281] [Medline: 32209532]

53. Pho AT, Bakken S, Lunn MR, et al. Online health information seeking, health literacy, and human papillomavirus vaccination among transgender and gender-diverse people. J Am Med Inform Assoc. Jan 12, 2022;29(2):285-295. [doi: 10.1093/jamia/ocab150] [Medline: 34383916]

54. Jiao W, Chang A, Ho M, Lu Q, Liu MT, Schulz PJ. Predicting and empowering health for generation Z by comparing health information seeking and digital health literacy: cross-sectional questionnaire study. J Med Internet Res. Oct 30, 2023;25:e47595. [doi: 10.2196/47595] [Medline: 37902832]

55. Robinson-Whelen S, Hughes RB, Alhusen JL, Beers L, Minard CG, Davidson D. Health information seeking in the digital age: a national survey of women with disabilities. Disabil Rehabil. Aug 2023;45(17):2751-2760. [doi: 10.1080/09638288.2022.2105960] [Medline: 35916449]

56. KHademian F, Arshadi Montazer MR, Aslani A. Web-based health information seeking and eHealth literacy among college students. A self-report study. Invest Educ Enferm. Feb 2020;38(1):e08. [doi: 10.17533/udea.iee.v38n1e08] [Medline: 32124576]

57. Htet H, Wichaidit W, Sriplung H, et al. Do electronic health literacy and online health information-seeking behavior mediate the effects of socio-demographic factors on COVID-19- and non-communicable disease-related behaviors among Myanmar migrants in Southern Thailand? Cureus. Nov 2023;15(11):e49090. [doi: 10.7759/cureus.49090] [Medline: 38125220]

58. Kim S, Park K, Jo HS. Gap between perceived eHealth literacy and ability to use online cancer-related information. J Korean Med Sci. Jun 22, 2020;35(24):e187. [doi: 10.3346/jkms.2020.35.e187]

59. Lee K, Hoti K, Hughes JD, Emmerton LM. Consumer use of “Dr Google”: a survey on health information-seeking behaviors and navigational needs. J Med Internet Res. Dec 29, 2015;17(12):e288. [doi: 10.2196/jmir.4345] [Medline: 26715363]

60. Stellefson ML, Shuster JJ, Chaney BH, et al. Web-based health information seeking and eHealth literacy among patients living with chronic obstructive pulmonary disease (COPD). Health Commun. Dec 2018;33(12):1410-1424. [doi: 10.1080/10410236.2017.1353868] [Medline: 28872905]

61. Peimani M, Stewart AL, Ghodssi-Ghassemabadi R, Nasli-Esfahani E, Ostovar A. The moderating role of e-health literacy and patient-physician communication in the relationship between online diabetes information-seeking behavior and self-care practices among individuals with type 2 diabetes. BMC Prim Care. Dec 30, 2024;25(1):442. [doi: 10.1186/s12875-024-02695-9] [Medline: 39736551]

62. Xie B. Older adults, e-health literacy, and collaborative learning: an experimental study. J Am Soc Inf Sci. May 2011;62(5):933-946. [doi: 10.1002/asi.21507]

63. Puspita N, Kurniawan AH, Tias CAN. Exploring the relationship between e-health literacy and online health information-seeking behaviour among pharmacy students in Indonesia. Pharm Educ. 2024;24(1):304-310. [doi: 10.46542/pe.2024.241.304310]

64. Ramstad KJ, Brørs G, Pettersen TR, et al. eHealth technology use and eHealth literacy after percutaneous coronary intervention. Eur J Cardiovasc Nurs. Jul 19, 2023;22(5):472-481. [doi: 10.1093/eurjcn/zvac087] [Medline: 36190843]
