# Supplementary figures and images for: The Relation Between eHealth Literacy and Online Health Information–Seeking Behavior: Systematic Review and Meta-Analysis
Source: J Med Internet Res. 2026 Jul 15;28:e93578. doi: 10.2196/93578 (PMC13372218; doi:10.2196/93578)

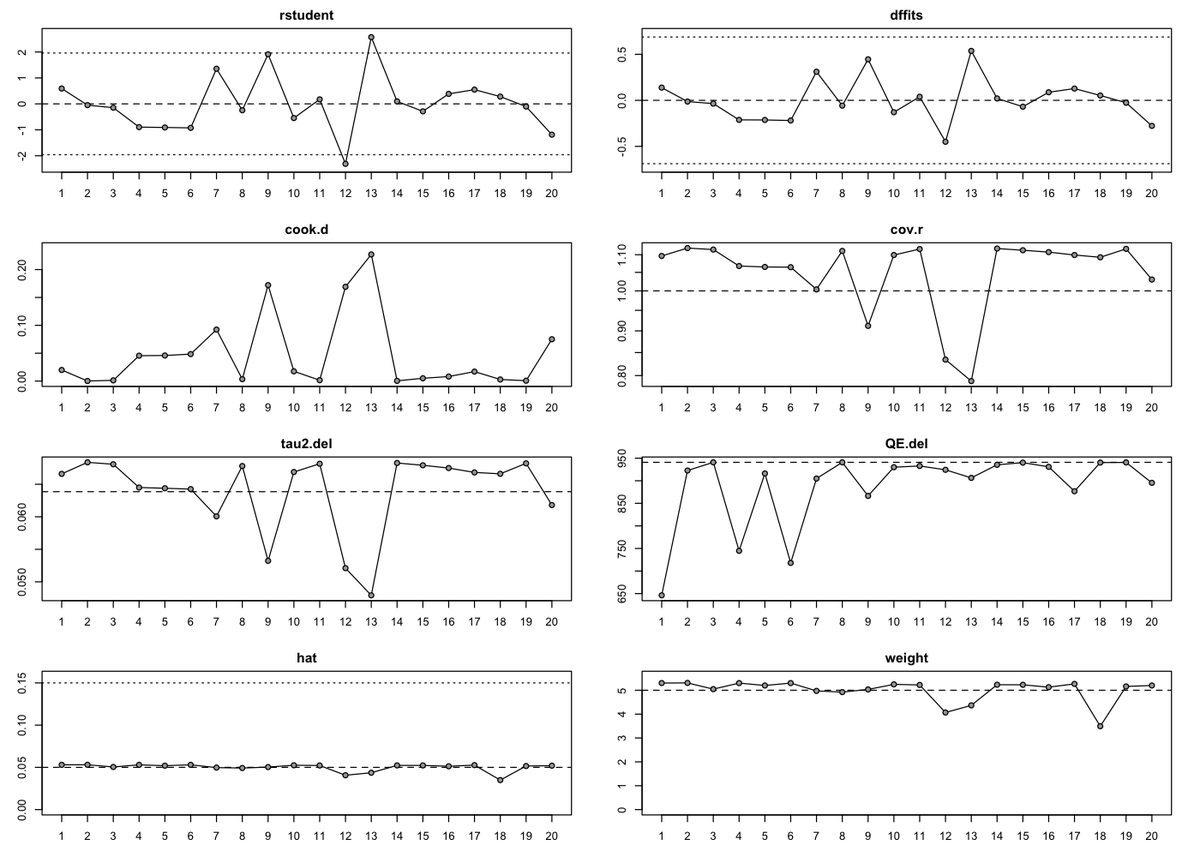

Supplement: Multimedia Appendix 5 [file jmir-v28-e93578-s005.png]
