# Supplementary material for: The Relation Between eHealth Literacy and Online Health Information–Seeking Behavior: Systematic Review and Meta-Analysis
Source: J Med Internet Res. 2026 Jul 15;28:e93578. doi: 10.2196/93578 (PMC13372218; doi:10.2196/93578)
Supplement: Multimedia Appendix 6 [file jmir-v28-e93578-s006.docx]

**Multimedia Appendix 6.** GRADE Summary of Findings for the association between eHealth literacy and online health information seeking behavior.

| **Outcome** | **k** | **Pooled r (95% CI)** | **Downgraded** | **Not downgraded** | **Certainty** |
| --- | --- | --- | --- | --- | --- |
| **Overall** | **20** | **0.27 (0.15–0.38)** | Inconsistency, imprecision | RoB, indirectness, pub. bias | ⊕⊖⊖⊖ Very low |
| Gen Z | 8 | 0.07 (−0.06–0.20) | Inconsistency, imprecision | RoB, indirectness, pub. bias | ⊕⊖⊖⊖ Very low |
| Non–Gen Z | 12 | 0.39 (0.27–0.50) | Inconsistency, imprecision | RoB, indirectness, pub. bias | ⊕⊖⊖⊖ Very low |
| Patients | 3 | 0.58 (0.01–0.86) | Inconsistency, imprecision | RoB, indirectness, pub. bias | ⊕⊖⊖⊖ Very low |
| Healthy | 17 | 0.22 (0.11–0.32) | Inconsistency, imprecision | RoB, indirectness, pub. bias | ⊕⊖⊖⊖ Very low |
| Professional sources | 5 | 0.41 (0.11–0.64) | Inconsistency, imprecision | RoB, indirectness, pub. bias | ⊕⊖⊖⊖ Very low |
| Non-professional sources | 14 | 0.21 (0.06–0.35) | Inconsistency, imprecision | RoB, indirectness, pub. bias | ⊕⊖⊖⊖ Very low |
