# Supplementary material for: The Relation Between eHealth Literacy and Online Health Information–Seeking Behavior: Systematic Review and Meta-Analysis
Source: J Med Internet Res. 2026 Jul 15;28:e93578. doi: 10.2196/93578 (PMC13372218; doi:10.2196/93578)
Supplement: Checklist 1 [file jmir-v28-e93578-s008.docx]

*From: Page MJ, McKenzie JE, Bossuyt PM, Boutron I, Hoffmann TC, Mulrow CD, et al. The PRISMA 2020 statement: an updated guideline for reporting systematic reviews. BMJ 2021;372:n71. doi: 10.1136/bmj.n71.*

| **Section and Topic** | **Item #** | **Items and elements recommended for reporting** | **Reported on** |
| --- | --- | --- | --- |
| **TITLE** | | | |
| **TITLE** | 1 | **Item: Identify the report as a systematic review.**  Elements:  • Identify the report as a systematic review in the title.  • Report an informative title that provides key information about the main objective or question the review addresses (e.g. the population(s) and intervention(s) the review addresses).  *• Consider providing additional information in the title, such as the method of analysis used, the designs of included studies, or an indication that the review is an update of an existing review, or a continually updated ("living") systematic review.* | Title, p.1 |
| **ABSTRACT** | | | |
| **ABSTRACT** | 2 | **Item: See the PRISMA 2020 for Abstracts checklist.**  Elements:  • Report an abstract addressing each item in the PRISMA 2020 for Abstracts checklist. | Abstract, p.1–3 |
| **INTRODUCTION** | | | |
| **RATIONALE** | 3 | **Item: Describe the rationale for the review in the context of existing knowledge.**  Elements:  • Describe the current state of knowledge and its uncertainties.  • Articulate why it is important to do the review.  *• If other systematic reviews addressing the same (or a largely similar) question are available, explain why the current review was considered necessary. If the review is an update or replication of a particular systematic review, indicate this and cite the previous review.*  *• If the review examines the effects of interventions, also briefly describe how the intervention(s) examined might work.*  *• If there is complexity in the intervention or context of its delivery (or both), consider presenting a logic model to visually display the hypothesised relationship between intervention components and outcomes.* | Introduction — Background, p.3–6 |
| **OBJECTIVES** | 4 | **Item: Provide an explicit statement of the objective(s) or question(s) the review addresses.**  Elements:  • Provide an explicit statement of all objective(s) or question(s) the review addresses, expressed in terms of a relevant question formulation framework.  *• If the purpose is to evaluate the effects of interventions, use the Population, Intervention, Comparator, Outcome (PICO) framework or one of its variants, to state the comparisons that will be made.* | Introduction — Objectives, p.6 |
| **METHODS** | | | |
| **ELIGIBILITY CRITERIA** | 5 | **Item: Specify the inclusion and exclusion criteria for the review and how studies were grouped for the syntheses.**  Elements:  • Specify all study characteristics used to decide whether a study was eligible for inclusion in the review, that is, components described in the PICO framework or one of its variants, and other characteristics, such as eligible study design(s) and setting(s), and minimum duration of follow-up.  • Specify eligibility criteria with regard to report characteristics, such as year of dissemination, language, and report status (e.g. whether reports, such as unpublished manuscripts and conference abstracts, were eligible for inclusion).  • Clearly indicate if studies were ineligible because the outcomes of interest were not measured, or ineligible because the results for the outcome of interest were not reported.  • Specify any groups used in the synthesis (e.g. intervention, outcome and population groups) and link these to the comparisons specified in the objectives (item #4).  *• Consider providing rationales for any notable restrictions to study eligibility.* | Methods — Eligibility Criteria, p.10–11 |
| **INFORMATION SOURCES** | 6 | **Item: Specify all databases, registers, websites, organisations, reference lists and other sources searched or consulted to identify studies. Specify the date when each source was last searched or consulted.**  Elements:  • Specify the date when each source (e.g. database, register, website, organisation) was last searched or consulted.  • If bibliographic databases were searched, specify for each database its name (e.g. MEDLINE, CINAHL), the interface or platform through which the database was searched (e.g. Ovid, EBSCOhost), and the dates of coverage (where this information is provided).  • If reference lists were examined, specify the types of references examined.  • If cited or citing reference searches (also called backward and forward citation searching) were conducted, specify the bibliographic details of the reports to which citation searching was applied, the citation index or platform used, and the date the citation searching was done. | Methods — Literature Search, p.8–9 |
| **SEARCH STRATEGY** | 7 | **Item: Present the full search strategies for all databases, registers and websites, including any filters and limits used.**  Elements:  • Provide the full line by line search strategy as run in each database with a sophisticated interface (such as Ovid), or the sequence of terms that were used to search simpler interfaces, such as search engines or websites.  • Describe any limits applied to the search strategy (e.g. date or language) and justify these by linking back to the review’s eligibility criteria.  *• If the search strategy was peer reviewed, report the peer review process used and specify any tool used such as the Peer Review of Electronic Search Strategies (PRESS) checklist.* | Methods — Search Strategy, p.9–10 + Multimedia Appendix 2 |
| **SELECTION PROCESS** | 8 | **Item: Specify the methods used to decide whether a study met the inclusion criteria of the review, including how many reviewers screened each record and each report retrieved, whether they worked independently, and if applicable, details of automation tools used in the process.**  Elements:  • Report how many reviewers screened each record (title/abstract) and each report retrieved, whether multiple reviewers worked independently at each stage of screening or not, and any processes used to resolve disagreements between screeners.  • Report any processes used to obtain or confirm relevant information from study investigators.  *• If abstracts or articles required translation into another language to determine their eligibility, report how these were translated.* | Methods — Selection Process, p.11 |
| **DATA COLLECTION PROCESS** | 9 | **Item: Specify the methods used to collect data from reports, including how many reviewers collected data from each report, whether they worked independently, any processes for obtaining or confirming data from study investigators, and if applicable, details of automation tools used in the process.**  Elements:  • Report how many reviewers collected data from each report, whether multiple reviewers worked independently or not, and any processes used to resolve disagreements between data collectors.  • Report any processes used to obtain or confirm relevant data from study investigators.  *• If any software was used to extract data from figures, specify the software used.* | Methods — Data Collection Process, p.11–12 |
| **DATA ITEMS (outcomes)** | 10a | **Item: List and define all outcomes for which data were sought. Specify whether all results that were compatible with each outcome domain in each study were sought, and if not, the methods used to decide which results to collect.**  Elements:  • List and define the outcome domains and time frame of measurement for which data were sought.  • Specify whether all results that were compatible with each outcome domain in each study were sought, and if not, what process was used to select results within eligible domains.  • If any changes were made to the inclusion or definition of the outcome domains, or to the importance given to them in the review, specify the changes, along with a rationale. | Methods — Operational Definitions of Key Constructs, p.7–8; Methods — Data Collection Process, p.11–12 |
| **DATA ITEMS (other variables)** | 10b | **Item: List and define all other variables for which data were sought (e.g. participant and intervention characteristics, funding sources). Describe any assumptions made about any missing or unclear information.**  Elements:  • List and define all other variables for which data were sought (e.g. participant and intervention characteristics, funding sources).  • Describe any assumptions made about any missing or unclear information from the studies.  *• If a tool was used to inform which data items to collect, cite the tool used.* | Methods — Data Collection Process, p.11–12 |
| **STUDY RISK OF BIAS ASSESSMENT** | 11 | **Item: Specify the methods used to assess risk of bias in the included studies, including details of the tool(s) used, how many reviewers assessed each study and whether they worked independently, and if applicable, details of automation tools used in the process.**  Elements:  • Specify the tool(s) (and version) used to assess risk of bias in the included studies.  • Specify the methodological domains/components/items of the risk of bias tool(s) used.  • Report whether an overall risk of bias judgement that summarised across domains/components/items was made, and if so, what rules were used to reach an overall judgement.  • Report how many reviewers assessed risk of bias in each study, whether multiple reviewers worked independently and any processes used to resolve disagreements between assessors. | Methods — Study Risk of Bias Assessment, p.12–13 |
| **EFFECT MEASURES** | 12 | **Item: Specify for each outcome the effect measure(s) (e.g. risk ratio, mean difference) used in the synthesis or presentation of results.**  Elements:  • Specify for each outcome (or type of outcome [e.g. binary, continuous]), the effect measure(s) (e.g. risk ratio, mean difference) used in the synthesis or presentation of results.  • State any thresholds (or ranges) used to interpret the size of effect (e.g. minimally important difference; ranges for no/trivial, small, moderate and large effects) and the rationale for these thresholds.  *• If synthesized results were re-expressed to a different effect measure, report the method used to re-express results.*  *• Consider providing justification for the choice of effect measure.* | Methods — Data Synthesis and Statistical Analysis, p.13–14 |
| **SYNTHESIS METHODS (eligibility for synthesis)** | 13a | **Item: Describe the processes used to decide which studies were eligible for each synthesis (e.g. tabulating the study intervention characteristics and comparing against the planned groups for each synthesis (item #5)).**  Elements:  • Describe the processes used to decide which studies were eligible for each synthesis. | Methods — Data Synthesis and Statistical Analysis, p.13 |
| **SYNTHESIS METHODS (preparing for synthesis)** | 13b | **Item: Describe any methods required to prepare the data for presentation or synthesis, such as handling of missing summary statistics, or data conversions.**  Elements:  • Report any methods required to prepare the data collected from studies for presentation or synthesis, such as handling of missing summary statistics, or data conversions. | Methods — Operational Definitions / Data Synthesis, p.7–8, p.13 |
| **SYNTHESIS METHODS (tabulation and graphical methods)** | 13c | **Item: Describe any methods used to tabulate or visually display results of individual studies and syntheses.**  Elements:  • Report chosen tabular structure(s) used to display results of individual studies and syntheses, along with details of the data presented.  • Report chosen graphical methods used to visually display results of individual studies and syntheses.  *• If studies are ordered or grouped within tables or graphs based on study characteristics (e.g. by size of the study effect, year of publication), consider reporting the basis for the chosen ordering/grouping.* | Methods — Data Synthesis and Statistical Analysis, p.13–15; Figures 3–5; Multimedia Appendix 3 |
| **SYNTHESIS METHODS (statistical synthesis methods)** | 13d | **Item: Describe any methods used to synthesize results and provide a rationale for the choice(s). If meta-analysis was performed, describe the model(s), method(s) to identify the presence and extent of statistical heterogeneity, and software package(s) used.**  Elements:  • If statistical synthesis methods were used, reference the software, packages and version numbers used to implement synthesis methods.  • If meta-analysis was done, specify: (a) the meta-analysis model (random-effects) and provide rationale for the selected model; (b) the method used (e.g. inverse-variance); (c) any methods used to identify or quantify statistical heterogeneity (e.g. τ², I², and prediction intervals).  • If a random-effects meta-analysis model was used: (a) specify the between-study (heterogeneity) variance estimator used (e.g. DerSimonian and Laird, restricted maximum likelihood (REML)); (b) specify the method used to calculate the confidence interval for the summary effect (e.g. Hartung-Knapp-Sidik-Jonkman). | Methods — Data Synthesis and Statistical Analysis, p.13–15 |
| **SYNTHESIS METHODS (methods to explore heterogeneity)** | 13e | **Item: Describe any methods used to explore possible causes of heterogeneity among study results (e.g. subgroup analysis, meta-regression).**  Elements:  • If methods were used to explore possible causes of statistical heterogeneity, specify the method used (e.g. subgroup analysis, meta-regression).  • If subgroup analysis or meta-regression was performed, specify for each: (a) which factors were explored, levels of those factors, and which direction of effect modification was expected and why; (b) whether analyses were conducted using study-level variables. | Methods — Subgroup and Sensitivity Analyses, p.15–16 |
| **SYNTHESIS METHODS (sensitivity analyses)** | 13f | **Item: Describe any sensitivity analyses conducted to assess robustness of the synthesized results.**  Elements:  • If sensitivity analyses were performed, provide details of each analysis (e.g. removal of studies at high risk of bias, use of an alternative meta-analysis model).  *• If any sensitivity analyses were not pre-specified, identify them as such.* | Methods — Subgroup and Sensitivity Analyses, p.15–16; Methods — Heterogeneity and Bias Assessment, p.14–15 |
| **REPORTING BIAS ASSESSMENT** | 14 | **Item: Describe any methods used to assess risk of bias due to missing results in a synthesis (arising from reporting biases).**  Elements:  • Specify the methods (tool, graphical, statistical or other) used to assess the risk of bias due to missing results in a synthesis (arising from reporting biases).  • If risk of bias due to missing results was assessed using an existing tool, specify the methodological components/domains/items of the tool, and the process used to reach a judgement of overall risk of bias. | Methods — Heterogeneity and Bias Assessment, p.14–15 |
| **CERTAINTY ASSESSMENT** | 15 | **Item: Describe any methods used to assess certainty (or confidence) in the body of evidence for an outcome.**  Elements:  • Specify the tool or system (and version) used to assess certainty (or confidence) in the body of evidence.  • Report the factors considered (e.g. precision of the effect estimate, consistency of findings across studies) and the criteria used to assess each factor when assessing certainty in the body of evidence.  • Describe the decision rules used to arrive at an overall judgement of the level of certainty, together with the intended interpretation (or definition) of each level of certainty.  • Report how many reviewers assessed certainty in the body of evidence for an outcome, whether multiple reviewers worked independently and any processes used to resolve disagreements between assessors. | Methods — Certainty of Evidence Assessment, p.15 |
| **RESULTS** | | | |
| **STUDY SELECTION (flow of studies)** | 16a | **Item: Describe the results of the search and selection process, from the number of records identified in the search to the number of studies included in the review, ideally using a flow diagram.**  Elements:  • Report, ideally using a flow diagram, the number of: records identified; records excluded before screening; records screened; records excluded after screening titles or titles and abstracts; reports retrieved for detailed evaluation; reports that did not meet inclusion criteria and the primary reasons for exclusion; and the number of studies and reports included in the review.  *• If applicable, indicate in the PRISMA flow diagram how many records were excluded by a human and how many by automation tools.* | Results — Study Selection, p.15–16 + Figure 2 |
| **STUDY SELECTION (excluded studies)** | 16b | **Item: Cite studies that might appear to meet the inclusion criteria, but which were excluded, and explain why they were excluded.**  Elements:  • Cite studies that might appear to meet the inclusion criteria, but which were excluded, and explain why they were excluded. | Results — Study Selection, p.15–16 + Figure 2 (exclusion reasons) |
| **STUDY CHARACTERISTICS** | 17 | **Item: Cite each included study and present its characteristics.**  Elements:  • Cite each included study.  • Present the key characteristics of each study in a table or figure (considering a format that will facilitate comparison of characteristics across the studies). | Results — Characteristics of the Included Studies, p.16–17 + Multimedia Appendix 3 |
| **RISK OF BIAS IN STUDIES** | 18 | **Item: Present assessments of risk of bias for each included study.**  Elements:  • Present tables or figures indicating for each study the risk of bias in each domain/component/item assessed (e.g. blinding of outcome assessors, missing outcome data) and overall study-level risk of bias.  • Present justification for each risk of bias judgement, for example in the form of relevant quotations from reports of included studies. | Results — Risk of Bias Assessment, p.17 + Multimedia Appendix 4 |
| **RESULTS OF INDIVIDUAL STUDIES** | 19 | **Item: For all outcomes, present, for each study: (a) summary statistics for each group (where appropriate) and (b) an effect estimate and its precision (e.g. confidence/credible interval), ideally using structured tables or plots.**  Elements:  • For all outcomes, irrespective of whether statistical synthesis was undertaken, present for each study summary statistics for each group (where appropriate).  • For all outcomes, irrespective of whether statistical synthesis was undertaken, present for each study an effect estimate and its precision (e.g. standard error or 95% confidence/credible interval).  *• If applicable, indicate which results were not reported directly and had to be computed or estimated from other information.* | Results — Quantitative Analysis, p.18–20 + Figure 3 + Multimedia Appendix 5 |
| **RESULTS OF SYNTHESES (characteristics of contributing studies)** | 20a | **Item: For each synthesis, briefly summarise the characteristics and risk of bias among contributing studies.**  Elements:  • Provide a brief summary of the characteristics and risk of bias among studies contributing to each synthesis (meta-analysis or other).  • Indicate which studies were included in each synthesis (e.g. by listing each study in a forest plot or table or citing studies in the text). | Results — Characteristics + Risk of Bias Assessment, p.16–17 + Multimedia Appendix 3, 4 |
| **RESULTS OF SYNTHESES (results of statistical syntheses)** | 20b | **Item: Present results of all statistical syntheses conducted. If meta-analysis was done, present for each the summary estimate and its precision (e.g. confidence/credible interval) and measures of statistical heterogeneity. If comparing groups, describe the direction of the effect.**  Elements:  • Report results of all statistical syntheses described in the protocol and all syntheses conducted that were not pre-specified.  • If meta-analysis was conducted, report for each: (a) the summary estimate and its precision (e.g. standard error or 95% confidence/credible interval); (b) measures of statistical heterogeneity (e.g. τ², I², prediction interval). | Results — Quantitative Analysis, p.18–21 + Table 1 + Figure 3 |
| **RESULTS OF SYNTHESES (results of investigations of heterogeneity)** | 20c | **Item: Present results of all investigations of possible causes of heterogeneity among study results.**  Elements:  • If investigations of possible causes of heterogeneity were conducted: (a) present results regardless of the statistical significance, magnitude, or direction of effect modification; (b) identify the studies contributing to each subgroup; (c) report results with due consideration to the observational nature of the analysis and risk of confounding due to other factors.  • If subgroup analysis was conducted: report for each analysis the exact P value for a test for interaction, as well as, within each subgroup, the summary estimates, their precision, and measures of heterogeneity. | Results — Quantitative Analysis (subgroup results), p.18–20 + Table 1 |
| **RESULTS OF SYNTHESES (results of sensitivity analyses)** | 20d | **Item: Present results of all sensitivity analyses conducted to assess the robustness of the synthesized results.**  Elements:  • If any sensitivity analyses were conducted: (a) report the results for each sensitivity analysis; (b) comment on how robust the main analysis was given the results of all corresponding sensitivity analyses. | Results — Sensitivity Analysis, p.20–22 + Multimedia Appendix 6 |
| **REPORTING BIASES** | 21 | **Item: Present assessments of risk of bias due to missing results (arising from reporting biases) for each synthesis assessed.**  Elements:  • Present assessments of risk of bias due to missing results (arising from reporting biases) for each synthesis assessed.  • If a funnel plot was generated to evaluate small-study effects, present the plot and specify the effect estimate and measure of precision used in the plot.  • If a test for funnel plot asymmetry was used, report the exact P value observed for the test, and potentially other relevant statistics, for example the standardised normal deviate, from which the P value is derived.  *• If any sensitivity analyses seeking to explore the potential impact of missing results on the synthesis were conducted, present results of each analysis.* | Results — Publication Bias Assessment, p.21 + Figures 4–5 |
| **CERTAINTY OF EVIDENCE** | 22 | **Item: Present assessments of certainty (or confidence) in the body of evidence for each outcome assessed.**  Elements:  • Report the overall level of certainty (or confidence) in the body of evidence for each important outcome.  • Provide an explanation of reasons for rating down (or rating up) the certainty of evidence (e.g. in footnotes to an evidence summary table).  *• Communicate certainty in the evidence wherever results are reported (i.e. abstract, evidence summary tables, results, conclusions), using a format appropriate for the section of the review.*  *• Consider including evidence summary tables, such as GRADE Summary of Findings tables.* | Results — Certainty of Evidence, p.25 + Multimedia Appendix 7 |
| **DISCUSSION** | | | |
| **DISCUSSION (interpretation)** | 23a | **Item: Provide a general interpretation of the results in the context of other evidence.**  Elements:  • Provide a general interpretation of the results in the context of other evidence. | Discussion — Prior Work and Principal Findings, p.25–27 |
| **DISCUSSION (limitations of evidence)** | 23b | **Item: Discuss any limitations of the evidence included in the review.**  Elements:  • Discuss any limitations of the evidence included in the review. | Discussion — Strengths and Limitations, p.30–31 |
| **DISCUSSION (limitations of review processes)** | 23c | **Item: Discuss any limitations of the review processes used.**  Elements:  • Discuss any limitations of the review processes used, and comment on the potential impact of each limitation. | Discussion — Strengths and Limitations, p.30–31 |
| **DISCUSSION (implications)** | 23d | **Item: Discuss implications of the results for practice, policy, and future research.**  Elements:  • Discuss implications of the results for practice and policy.  • Make explicit recommendations for future research. | Discussion — Implications throughout discussion subsections, p.27–30; Conclusion, p.31–32 |
| **OTHER INFORMATION** | | | |
| **REGISTRATION AND PROTOCOL (registration)** | 24a | **Item: Provide registration information for the review, including register name and registration number, or state that the review was not registered.**  Elements:  • Provide registration information for the review, including register name and registration number, or state that the review was not registered. | Abstract, p.2; Methods — Literature Search, p.8–9 (PROSPERO CRD420251088300) |
| **REGISTRATION AND PROTOCOL (protocol)** | 24b | **Item: Indicate where the review protocol can be accessed, or state that a protocol was not prepared.**  Elements:  • Indicate where the review protocol can be accessed (e.g. by providing a citation, DOI or link), or state that a protocol was not prepared. | Methods — Literature Search, p.9 (PROSPERO website) |
| **REGISTRATION AND PROTOCOL (amendments)** | 24c | **Item: Describe and explain any amendments to information provided at registration or in the protocol.**  Elements:  • Report details of any amendments to information provided at registration or in the protocol, noting: (a) the amendment itself; (b) the reason for the amendment; and (c) the stage of the review process at which the amendment was implemented. | N/A — no amendments |
| **SUPPORT** | 25 | **Item: Describe sources of financial or non-financial support for the review, and the role of the funders or sponsors in the review.**  Elements:  • Describe sources of financial or non-financial support for the review, specifying relevant grant ID numbers for each funder. If no specific financial or non-financial support was received, this should be stated.  • Describe the role of the funders or sponsors (or both) in the review. If funders or sponsors had no role in the review, this should be declared. | Funding, p.32 |
| **COMPETING INTERESTS** | 26 | **Item: Declare any competing interests of review authors.**  Elements:  • Disclose any of the authors’ relationships or activities that readers could consider pertinent or to have influenced the review. | Conflicts of Interest, p.33 |
| **AVAILABILITY OF DATA, CODE, AND OTHER MATERIALS** | 27 | **Item: Report which of the following are publicly available and where they can be found: template data collection forms; data extracted from included studies; data used for all analyses; analytic code; any other materials used in the review.**  Elements:  • Report which of the following are publicly available: template data collection forms; data extracted from included studies; data used for all analyses; analytic code; any other materials used in the review.  • If any of the above materials are publicly available, report where they can be found (e.g. provide a link to files deposited in a public repository).  *• If data, analytic code, or other materials will be made available upon request, provide the contact details of the author responsible for sharing the materials and describe the circumstances under which such materials will be shared.* | Data Availability, p.33 |
